# A hybrid demand-side policy for balanced economic emission in microgrid systems

**DOI:** 10.1016/j.isci.2025.112121

**Published:** 2025-02-27

**Authors:** Arvind R. Singh, Bishwajit Dey, Srikant Misra, Rangu Seshu Kumar, Mohit Bajaj, Vojtech Blazek

**Affiliations:** 1 Department of Electrical Engineering, School of Physics and Electronic Engineering, Hanjiang Normal University, Shiyan, China; 2 Department of Electrical Engineering, Manipal University, Jaipur, Rajasthan, India; 3 Department of Electrical and Electronics Engineering, GIET University, Gunupur, Odisha, India; 4 Department of Electrical and Electronics Engineering, Vignan’s Foundation for Science Technology and Research (Deemed to be University), Vadlamudi, Guntur 522213, India; 5 Department of Electrical Engineering, Graphic Era (Deemed to be University), Dehradun 248002, India; 6 Hourani Center for Applied Scientific Research, Al-Ahliyya Amman University, Amman, Jordan; 7 College of Engineering, University of Business and Technology, Jeddah 21448, Saudi Arabia; 8 ENET Centre, CEET, VSB-Technical University of Ostrava, 708 00 Ostrava, Czech Republic

**Keywords:** Energy engineering, Energy Resources, Energy systems, Environmental policy

## Abstract

Demand-side management (DSM) enhances distribution network efficiency by shifting or reducing loads, alleviating network stress. The Load Shifting Policy (LSP) reallocates flexible loads to low-price periods without altering total demand, while the Load Curtailing Policy (LCP) incentivizes consumers to reduce peak demand. This study introduces a hybrid DSM approach that combines LSP and LCP with a smart charging strategy for plug-in hybrid electric vehicles (PHEVs). Using the hybrid load shifting and curtailment policy (HLSCP), the microgrid (MG) load profile was optimized, reducing generation costs from 707¥ for the base load to 682¥ with HLSCP and 676¥ when incorporating smart PHEV charging. Emissions decreased correspondingly, from 1267kg to 1246kg. These results demonstrate the hybrid DSM’s capacity to tackle economic and environmental challenges in power systems. The Differential Evolution (DE) optimization method further validated the robustness and efficiency of this cost-effective, sustainable microgrid management approach.

## Highlights

• Hybrid DSM integrates load shifting and curtailment for microgrid optimization
• Smart PHEV charging reduces emissions and operational costs in MG systems
• Proposed strategies achieve a 20% cost reduction and 10% emission decrease
• Differential Evolution algorithm ensures robust optimization for DSM strategies

## Graphical abstract

**Figure:**
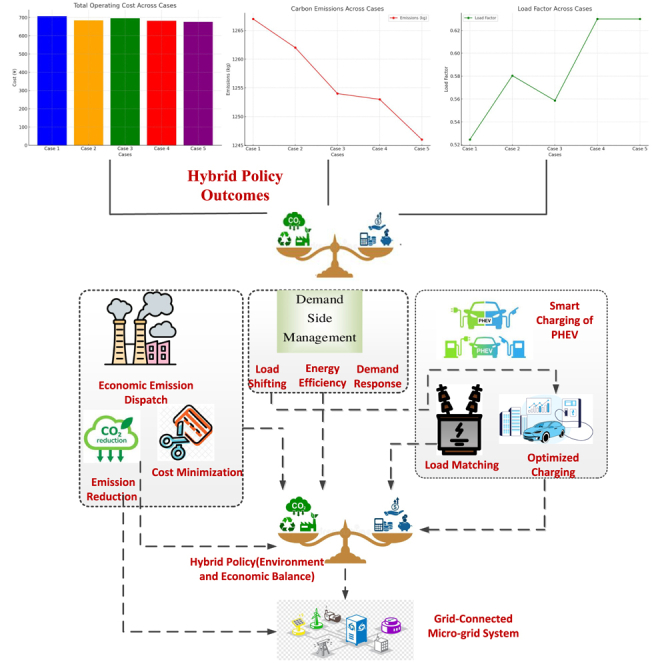

## Introduction

### Background

A microgrid (MG) is a small-scale distribution system that incorporates distributed generators (DGs) and engaged consumers, with the ability to link to the main grid. Microgrids may enable the incorporation of renewable energy sources, plug-in electric vehicles (PEVs), and plug-in hybrid electric vehicles (PHEVs) to alleviate the worldwide concern about environmental problems and climate change. Using MGs in power systems would provide many benefits, including reducing power losses resulting from the close proximity between the load and generation. Additionally, MGs would enhance power quality and alleviate operational expenses. Microgrids can operate either in sync with the main grid or independently from it, which are known as grid-connected and islanded modes, respectively. Incorporating these functionalities enhances system reliability and efficiency while reducing load shedding. RES are vital in ELD. The various entities interconnected in the network of the power system cannot supply the load demand with the same quantity of power they generate, as drawn from ELD. Instead, they partially compensate for this by adjusting their energy prices.

**Figure 1:**
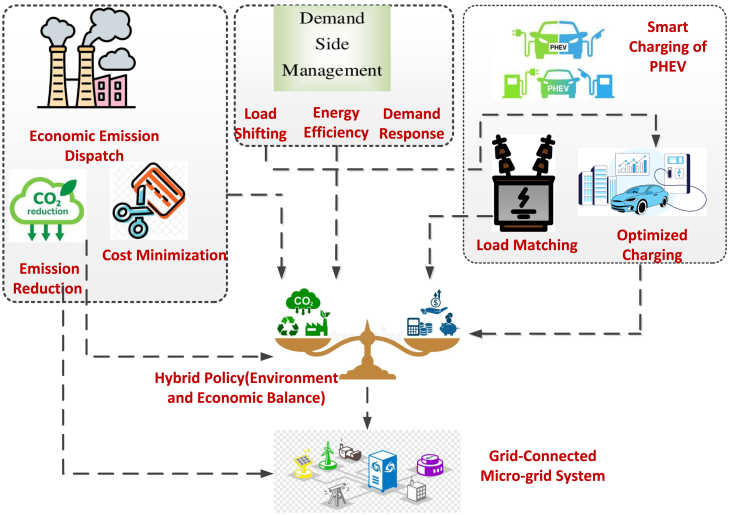
Typical Microgrid structure with the integration of DSM programs

The amount of active power produced per unit of output may differ among devices. Nevertheless, the total power generation must equal the overall demand. In this context, demand-side management (DSM) plays a crucial role in optimizing MG performance. DSM strategies, such as load shifting and curtailment, not only balance generation and demand but also help achieve economic and environmental objectives. This research work integrates a hybrid DSM approach with smart PHEV charging to further optimize MG operations, tackling the dual challenges of cost efficiency and emission reduction. The framework developed to achieve these objectives is depicted in Figure 1.

### Literature review

In recent years, renewable energy sources (RESs) have garnered increased attention from researchers and engineers due to their potential to reduce reliance on fossil fuels, thereby lowering pollutant emissions. Additionally, they contribute to enhanced power quality, reduced power losses, and numerous environmental benefits. RESs are clean energy options that are relatively simple to install. However, despite these advantages, the integration of such resources into electrical distribution systems can introduce several challenges. Specifically, the high penetration of RESs can complicate the processes of protection, operation, and control within distribution networks. To address these issues, the microgrid (MG) concept has been proposed as a solution. MGs combine various distributed generators (DGs) with controllable loads to create a more manageable system. This technology enables more effective integration of grid-connected RESs for large-scale applications, making an energy management strategy (EMS) essential to ensure the optimal use of DGs in a coordinated, intelligent, and secure manner. Consequently, many researchers have focused on developing EMS approaches that facilitate the optimal operation of MGs, with the primary objectives of minimizing operating costs and reducing environmental impact. The improvement of MG performance has thus become a key area of research. Hai, Tao et al.^1^ introduces a distinctive method for addressing the problem, focusing on minimizing reliability costs to achieve the lowest total grid cost. Simultaneously, the transportation sector has been gradually replacing conventional vehicles with EVs, PEVs, and PHEVs, generating significant public interest. Fathy, Ahmed^2^ introduced an innovative energy management strategy that incorporates the Bald Eagle Search (BES) optimizer for microgrids integrated with renewable energy sources and PHEVs. This approach is designed to control and optimize the production of each unit within the microgrid. This is done by framing the subject as an optimization problem to lower the overall operational cost and reduce the discharge of environmental pollutants. A comparative study among the ED, Emission dispatch, CEED based on fractional programming (FP), and ECED has been discussed by Chhualsingh, Tapas et al.^3^ Zheng, Yangbing et al.^4^ research work underlined the intrinsic problems of MGs, RES, and PHEVs and proposed a new direction toward a sustainable energy future. The main contribution consists of an architecture of multi-stage dynamic EM, especially developed to maximize the utilization of non-dispatchable RESs and PHEVs while minimizing reliance on the UG. Hassan, Arfan M. Salih^5^ presented an integrated optimization paradigm for MG resource scheduling, considering EVs and demand-side management. While wind and solar energy sources contribute only a portion of the overall consumption demand, the microgrid remains connected to the main electrical grid to facilitate power exchange. This process determines the scheduling optimization of a microgrid containing multiple PHEVs and RESs for economic, technological, and environmental benefits. The Mont Carlo simulation (MCS) simulates the intermittent characteristics of renewable resources, PHEVs, and loads to improve the microgrid operation precision discussed by Hai, Tao et al.^6^ Alghamdi, Ali S.^7^ proposed a new approach to enhance the energy coordination and microgrid activity scheduling. Wang, Y. et al.^8^ presented technique has focused on implementing an IGBO algorithm that results in higher renewable energy production and lower energy running costs. The research work introduced the improvement in the microgrids management by integrating multi-energy microgrids with the latest technology such as electric vehicles. The considered MEM demand response schemes in this under energy sources include wind energy, multi-carrier energy storage technologies, boilers, combined heat and cooling units, electric vehicles (EV), power-to-gas systems, and mechanisms responding to demand. Chakraborty A. et al.^9^ developed a stochastic expert technique to minimize overall operating cost for a grid-connected low-voltage microgrid, taking into account the charging effect of plug-in hybrid electric vehicles and giving the optimum appropriate size of the BESS. Integrating PHEV into an energy hub enhances batteries to work as a versatile storage system, which enhances the potential integration of RES in electrical power system networks. Zhang, T. et al.^10^ proposed a new model for estimating the uncertainty of the PHEV consumption during travel. The model is based on the information gap decision-making theory, using strategies of risk aversion and risk-taking. Hai, Tao et al.^11^ proposed an ideal management strategy for a microgrid including renewable sources and electric vehicles together with responsive loads, is supposed to minimize operating costs as well as emissions. While under high-load circumstances, electric vehicles play the major role, the reactive loads are employed for countering the uncertainties related to renewable-based sources. Yang, Z. et al.^12^ presented the research article for the optimum energy-heat programming of a stochastic model and daily storage of a microgrid. In that respect, the approach of a bi-level stochastic programming is initiated toward integrated energy-heat scheduling and storage optimization in the existence of an ESS and DR model with a view to maximize societal welfare. A decentralized energy management system for DC microgrid, by incorporating the RTP program proposed by Singh, A. et al.^13^ In this scheme, the sectoral connection between conventional electrical energy and hydrogen energy is considered. Integrating electric vehicles with reactive loads for power consumption in a microgrid to optimize operating expenses and resultant emissions, taking into consideration wind and solar variable sources discussed by Ali, Ziad M. et al.^14^ For this purpose, the proposed methodology will use electric vehicles as a way to reduce peak demand, shifting the load curve. Plug-in electric vehicles may potentially destabilize the electrical grid, thus deteriorating system performance. Hai Tao et al.^15^ developed the probabilistic optimization model to manage the charging mechanism of PEVs for generating and storage assets. Bokopane, Lindiwe et al.^16^ was focused on to enhance the system’s reliability and profitability through operational cost reductions. Armioun, Majid et al.^17^ presented a new two-level and two-step optimization approach to effectively clear the multicarrier energy distribution systems and microgrids, both for day-ahead and real-time scenarios. The incentive-and price-based demand response schemes are included in the model to encourage participation by microgrids with the distribution system. A hybrid approach to reduce operating costs in networked microgrids was outlined by Annamalai, T. et al.^18^ Roy, Nibir Baran al al.^19^ implemented a hybrid model enhances the power quality for an islanded microgrid in the presence of DERs respectively. While dispersion sources, such as renewable-based distributed generation are integrated into medium voltage distribution networks, coordination in operation and management becomes difficult because of the time-varying load with intermittent characteristics for both distribution networks and microgrid operators was discussed by Singh, Arvind R. et al.^20^ For a multi-carrier zero bus microgrid, a sequential stochastic coordinated energy management scheme has been applied in the presence of renewable sources, auxiliary boilers, controllable loads, distributed tri-generation plants, energy storage systems, chiller units, and plug-in hybrid electric vehicles by Roy, Nibir Baran al al.^21^ A multi-objective optimization model is developed in order to maximize goals such as pollution emission, operation cost, and loss of load expectation (LOLE) with respect to renewable energy sources (RES) given by Alzahrani, Ahmad et al.^22^ This model uses a non-dominated genetic sorting method. Beta probability density function (PDF) is used in describing RES as being intermittent and unreliable—for example, wind and sun. In the research article, the stochastic optimum operation was proposed for an MG composed of a large number of RES, storage systems, and plug-in hybrid electric vehicles. Ali Ziad M. et al.^23^ proposed the Monte Carlo simulation models for the uncertainties of the MG. M. Li et al.^24^ proposed a scheduling framework for electric vehicles and controlled loads for microgrid operational cost and pollution minimization. This microgrid generates renewable electricity using wind turbines and solar photovoltaic panels. A new avenue to minimize operational costs and determine the least cost-effective grid using the fewest resources was proposed by Hai, Tao et al.^25^ The transport sector is going with electric vehicles now coming in the form of PEVs and PHEVs, both of which are gaining increasing popularity. AL-Dhaifallah et al.^26^ investigated the impact of PHEV charging on the optimal operation of microgrids. To evaluate the PHEV performance, 3 different patterns for PHEV charging have been considered: uncontrolled, regulated, and smart charging ways. Sudhakar, A. et al.^27^ proposed a methodology to smoothen the intermittent characteristics of other renewable sources for effectively operating a microgrid acting as virtual storage. In this article, a tri-level energy management system involving Optimal storage distribution, power exchange, and intelligent EV ranking is proposed. Rajagopalan, Arul et al.^28^ has presented a novelty energy management approach to implement an iterative map-based self-adaptive crystal structure algorithm called SaCryStAl, which was dedicated to a microgrid integrated with renewable energy sources and plug-in hybrid electric vehicles. A flexible, distributed, and instantaneous power management system is postulated considering limitations of economic dispatch, reduction of overhead transmission lines, and techniques for self-healing failures outlined by Rahim, Sahar et al.^29^ The developed cutting-edge model is thus suitable for all categories of energy customers, residential, commercial and industrial, utility firms, and the power grid. Tao, Hai et al.^30^ proposed a very efficient day-ahead resource scheduling paradigm for an MG, which considers PHEVs and RESs. The model has been established with respect to a microgrid that is outfitted with both renewable and non-renewable energy-based DG technologies, storage devices, and plug-in hybrid electric vehicles. A multi-objective optimum dispatch model for a microgrid operating in the grid-connected mode, considering both the operational cost and the environmental protection cost of the microgrid system has been discussed by Lu, X. et al.^31^ A multi-objective optimum load dispatch model of the microgrid with the stochastic access of the electric vehicle has been proposed by Lu X. et al.^32^ Uncertainties of electric vehicles are modeled by Monte Carlo simulation. The optimum performance of a grid-connected MG is discussed under a DRP (RTP) mechanism by Barhagh, S. S. et al.^33^ In this article, the RC energy system is examined with an integrated connection of PV units and EVs that may contribute to the RCs in fulfilling their energy demand as well as offering economic advantages.

The integration of advanced energy management strategies and optimization techniques for renewable energy and electric vehicle (EV) systems has been a focal point in recent studies. Sabyasachi et al.^34^ explored a holistic business model for the EV charging ecosystem, emphasizing sustainability and operational efficiency, closely related to the optimization of demand-side management (DSM) for grid-connected microgrids. Kumar et al.^35^ introduced a hybrid genetic algorithm and simulated annealing approach for EV charging station placement, focusing on enhancing network resilience, a goal aligned with the Hybrid Load Shifting Curtailment Policy (HLSCP) for achieving both system robustness and cost-efficiency. Nagarajan et al.^36^ proposed a Cheetah-inspired algorithm for dynamic economic dispatch in renewable-integrated systems, emphasizing smart adaptive load management, which resonates with the implementation of smart charging strategies for PHEVs. Another important study by Kumar et al.^37^ detailed efficient and reliable EV charging strategies to foster sustainable transportation systems, underscoring the significance of cost-effective and reliable energy management solutions, objectives addressed by the hybrid DSM approach. Panda et al.^38^ conducted a comprehensive review of DSM and market design for renewable energy integration, identifying challenges and optimization frameworks. These insights provide a broader context to the specific DSM implementations, such as the load shifting and curtailment strategies. Similarly, Mohanty et al.^39^ surveyed strategies and challenges in EV demand-side management, offering modeling techniques that complement the incorporation of smart charging frameworks for PHEVs. Panda et al.^40^ further expanded on residential DSM models, discussing the future potential of hybrid optimization approaches, which align with the integration of load shifting and curtailing policies for energy management. Davoudkhani et al.^41^ introduced a mountaineering team-based optimization algorithm for load frequency control in microgrids, demonstrating the potential of novel algorithms for enhancing microgrid stability. This aligns with the application of the Differential Evolution (DE) algorithm for optimizing DSM strategies. Singh et al.^42^ utilized machine learning to enhance energy management and power forecasting in microgrids with distributed energy sources, showcasing advanced computational approaches for system reliability, a concept reflected in the robust optimization strategies of the study. Karthik et al.^43^ proposed a chaotic sine cosine optimization algorithm for multi-objective energy scheduling, emphasizing frameworks for balancing economic and environmental goals, similar to the balanced economic-emission objectives in energy management systems. Nadimuthu et al.^44^ investigated renewable energy microgrids with vehicle-to-grid (V2G) technology, demonstrating its potential for rural electrification, indirectly supporting the focus on energy cost reduction and grid stability through PHEVs. Panda et al.^45^ proposed a priority-based scheduling strategy using the adaptive salp swarm algorithm for residential energy systems, illustrating the utility of advanced heuristic optimization methods akin to the DE approach. Nagarajan et al.^46^ developed an enhanced wombat optimization algorithm for multi-objective power flow in renewable and EV-integrated systems, contributing to energy management strategies that align with the integrated approach to load management. Singh et al.^47^ further extended the use of this optimization framework, highlighting the importance of EV integration in achieving optimal power flow in renewable-integrated microgrids. These studies collectively offer a multi-dimensional understanding of the challenges and opportunities in renewable-integrated microgrids and EV systems.

The development of efficient demand-side management (DSM) strategies is a critical aspect of optimizing smart grid performance, with numerous studies addressing various facets of this challenge. Ma et al.^48^ explore the use of relaying-assisted communications for demand response (DR) in smart grids, presenting cost modeling, game strategies, and algorithms to effectively manage load fluctuations and optimize economic dispatch. Their work highlights the potential of advanced communication and game-theoretic approaches in reducing stress on the grid and enhancing load balancing, which is a fundamental aspect of DSM in modern energy systems. Yang et al.^49^ build on this concept by introducing a three-stage multi-energy trading strategy based on peer-to-peer (P2P) trading mechanisms, allowing decentralized energy transactions that help balance supply and demand in multi-energy systems. This approach is valuable for integrating diverse energy sources, including electricity, heat, and gas, into DSM frameworks, showcasing how market-based strategies can complement optimization efforts in smart grids. Similarly, Li et al.^50^ propose a two-stage economic-safety optimization model for hydrogen energy storage sizing, emphasizing the integration of storage systems in multi-energy networks. Their model accounts for both economic efficiency and safety considerations, highlighting the crucial role of energy storage in improving the flexibility and stability of power systems—an area that is essential for enhancing DSM strategies. In contrast, Zhang et al.^51^ and Zhang et al.^52^ address the resilience of distributed energy management systems under cyber-attacks, focusing on event-triggered mechanisms that enhance security. These studies emphasize the importance of safeguarding smart grids from external disruptions, ensuring that DSM strategies remain robust and reliable in real-world applications. On the optimization front, Zhang et al.^53^ apply deep reinforcement learning (DRL) for multi-objective optimization in microgrid dispatch, which includes both economic and emission dispatch. Their integration of DRL into energy management systems offers a promising approach to improving system adaptability, an essential characteristic for optimizing DSM policies. The hierarchical optimization strategy proposed by Chen et al.^54^ for integrated electricity-heat-ammonia microgrid clusters offers a sophisticated approach to managing multi-source energy systems. Their work provides a framework for handling the complexities of energy integration, which is critical for balancing economic objectives and reducing emissions in microgrids. Deng et al.^55^ explore the combination of distributed DC energy systems with deep reinforcement learning to optimize renewable energy operations in buildings, showcasing the potential of AI to enhance the operation of renewable systems in DSM contexts. This study contributes valuable insights into optimizing renewable energy generation and consumption, which is a key challenge for future DSM strategies in grid-connected systems. Zhang et al.^56^ investigate the techno-environmental-economic performance of allocating multiple energy storage resources for various urban forms, highlighting the importance of optimizing storage systems to achieve low-carbon goals. Their work is directly applicable to the optimization of energy storage in DSM systems, particularly in urban settings where space and resources are limited. Lastly, Feng et al.^57^ examine the role of automakers in developing optimal strategies for electric vehicle (EV) charging stations. Their research highlights the need for coordinated strategies to manage EV charging infrastructure, which is becoming increasingly important as EVs are integrated into smart grids. This work underscores the relevance of considering flexible loads, such as EVs, in DSM policies to enhance grid stability and reduce operational costs. Collectively, these studies provide comprehensive insights into the optimization of energy systems, offering a diverse range of strategies—ranging from decentralized energy trading and multi-energy integration to cybersecurity and advanced optimization techniques—that can significantly enhance the effectiveness of DSM in smart grids. A decarbonized microgrid system using the DR program was suggested with a day-ahead electricity market by Alhasnawi et al.^58^ Reducing operational costs and carbon emissions is the primary goal of the suggested system. By considering the present load demand, energy prices, and generating capacity, the suggested unit employs the African Vultures Optimization Algorithm (AVOA) to optimize the operating cost. Alhasnawi et al.^59^ presented an effective energy optimization method for Smart Urban Buildings (SUBs) using the Improved Sine Cosine Algorithm (ISCA) and employing load-shifting techniques for demand-side management to enhance energy consumption patterns in SUBs. The objective of the proposed system is to enhance the energy efficiency of SUB appliances to efficiently manage load demand, ultimately resulting in a decreased peak to average ratio (PAR) and a subsequent reduction in power expenses. Alhasnawi et al.^60^ present an efficient energy management strategy for the optimum management of the microgrid using sophisticated mixed-integer linear programming. This study proposes a demand-side management (DSM) engine using mixed-integer linear programming for an IoT-enabled grid. Alhasnawi et al.^61^ introduced a concept for a smart unit designed for the operation and cost management of multi-source microgrids. The suggested unit uses the Improved Artificial Rabbits Optimization Algorithm (IAROA) to optimize operational costs in relation to current load demand, energy prices, and generating capacity. Alhasnawi et al.^62^ optimize system design concerns that govern decision-making for optimal operation management with the Improved Crow Search Algorithm (ICSA). The simulation findings indicate that the proposed algorithm-driven ICSA decreases carbon emissions, peak-to-average ratio (PAR), and power expenses. Bakhshaei et al.^63^ examine the best acquisition of grid electricity in a grid-connected photovoltaic/pumped hydro storage (PV/PHS) system, taking into account a demand response program (DRP). The incentive rate of DRP is also determined to implement DRP effectively. Nehmedo et al.^64^ introduce a novel application of the Pelican Optimization Algorithm (POA) for optimum Energy Management (EM) in Microgrids (MG) with the consideration of the Demand Response Program (DRP). To optimize the benefits of the MG operator (MGO) and minimize total operating expenses, including traditional generator fuel costs and power transaction fees, a multi-objective optimization approach is established. Internal and external markets to facilitate MGs' successful involvement in energy trading, including energy exchanges between MGs and the utility grid (UG) have been examined by Datta et al.^65^ An energy management approach for multi-microgrid systems, focusing on the scheduling of distributed resources, renewable energy sources, and the integration of plug-in electric cars to optimize the economic and environmental advantages of the system has been formulated by Datta et al.^66^ Kujur et al.^67^ proposes Chaotic Aquila Optimization for addressing the demand response program of a grid-connected residential microgrid (GCRMG) system. The primary aim is to optimize the scheduling of interconnected appliances inside the building to reduce total user costs under fluctuating power prices. Chakraborty et al.^68^ introduce the slime mold algorithm (SMA) to address this intricate optimization challenge. Initially, SMA is evaluated across three scenarios, focusing on the reduction of operating costs and emissions as distinct goals. Chakraborty et al.^69^ seek to concurrently reduce the daily operating expenses and net environmental pollution of a small microgrid system, considering the charging requirements of Plug-in Hybrid Electric Vehicles (PHEVs) and consumer load needs. El-Bayeh et al.^70^ provide a detailed survey on the strategies for charging and discharging electric vehicles (EVs), focusing on the optimization of energy flow between EVs and the grid, highlighting the importance of effective load management to ensure grid stability and minimize battery wear. Venkatesh B. et al.^71^ investigate demand management in microgrids by implementing load shifting techniques, utilizing controllable devices and a hybrid optimization method (WFS2ACSO) to optimize energy distribution and improve grid reliability. López et al.^72^ explore demand-side management for smart charging of electric vehicles through deep learning algorithms, demonstrating how predictive models can optimize charging schedules, reducing peak demand and enhancing grid efficiency. Sankaramurthy et al.^73^ address power grid congestion by rescheduling generators and incorporating pumped hydro storage units, using a Flower Pollination Optimization (FPO) algorithm to optimize power generation scheduling and alleviate congestion in power systems. These studies focus on innovative strategies for optimizing energy management, charging, and grid operations with a particular emphasis on integrating electric vehicles and renewable energy sources.

### Motivation and contribution

Existing research on microgrid (MG) energy management has extensively explored individual demand response plans (DRPs) such as load shifting or load curtailment strategies to enhance economic energy management. Rearranging anticipated load demand to remove or shift flexible loads to periods when electricity is less expensive helps to improve the power generation economy and reduce utility dependence. Additionally, some studies have introduced a load reduction strategy where microgrid operators incentivize consumers to reduce their energy usage during peak hours, effectively encouraging better demand management through rewards. However, the possibility of integrating load shifting and curtailment measures simultaneously has not been comprehensively addressed in the existing literature. Most power system researchers, despite their thorough investigations, have overlooked combining these measures in a hybrid framework. This represents a significant research gap in achieving balanced economic and environmental objectives in MG systems. Furthermore, there is a lack of comprehensive techno-economic studies examining these strategies under carbon constraints, particularly in the context of plug-in hybrid electric vehicles (PHEVs) and smart charging systems, which are vital for optimizing load profiles and enhancing MG flexibility. In comparison to the existing state of the art, the current work contributes to the following advancements.

(1) The baseline load demand model was modified to incorporate load shifting, curtailment, and innovative hybrid strategies combining both load shifting and curtailment.
(2) In this research work for the different load demand models were assessed to determine key factors such as load factor, peak demand reduction, customer incentives, and energy savings. For each model, economic dispatch, emission dispatch, and a combined economic-emission weighted dispatch were carried out to comprehensively evaluate performance. The simulated results were thoroughly compared with findings from previous studies.

As highlighted in our literature review, previous studies primarily focused on implementing either load shifting or load curtailment strategies independently. The main aim of our proposed hybrid DSM strategy lies in its ability to combine both approaches, thereby addressing the limitations of individual policies. This hybrid implementation was further evaluated under economic dispatch (ED), emission dispatch (EMD), and balanced economic-emission dispatch (ECED) objectives, demonstrating superior performance compared to conventional methods. The adoption of the Differential Evolution (DE) algorithm ensures an efficient and robust optimization process for scheduling Distributed Energy Resources (DERs) and PHEVs under the hybrid DSM strategy. The results obtained show clear improvements in cost, emissions, and system reliability, validating the effectiveness of the proposed approach. The selection of the hybrid DSM strategies, which combine Load Shifting Policy (LSP) and Load Curtailing Policy (LCP), was motivated by the need to achieve a balanced and optimized trade-off between cost minimization, carbon emission reduction, and grid stability. Existing DSM strategies, such as LSP and LCP, are often implemented individually, leading to suboptimal results in terms of system-wide performance. The hybrid approach proposed in this study addresses this research gap by combining the benefits of both strategies, allowing for enhanced flexibility and performance improvements. The primary reasons are outlined later in discussion:

*Improvement in Load Factor and Peak Load Reduction****:*** The LSP effectively reduces peak loads by shifting elastic loads to off-peak hours without altering the overall load demand. This improves the load factor and reduces the stress on the grid. The LCP, on the other hand, encourages load reduction during peak hours by providing incentives to consumers, directly decreasing the energy demand. Combining these two strategies ensures a more robust and efficient load management approach that achieves both load flattening and energy savings.

*Economic and Environmental Benefits***:** The hybrid strategy (HLSCP) achieves significant cost savings for the grid operator while offering incentives to participating consumers, fostering higher participation rates. The curtailment of unnecessary loads, post load shifting, further reduces energy consumption, leading to a reduction in operational costs and carbon emissions.

*Synergistic Use of Smart Charging for PHEVs***:** By integrating smart charging strategies for Plug-in Hybrid Electric Vehicles (PHEVs) within the hybrid DSM framework, the system leverages the ability to optimize energy usage during off-peak hours. Smart charging not only enhances grid stability but also reduces overall energy costs by taking advantage of time-of-use pricing mechanisms.

Table 1 later in discussion discusses the contribution of some recently published articles in line with our work and points out the contribution of our work attends to the research gap among the others.

**Table 1:** Comparative literature survey to emphasis the novel contribution of this article

Optimization Algorithm	RES	ESS	LSP	LCP	Balanced economic emission dispatch	HDSM with balanced economic emission dispatch	Year	Ref. No
Improved Gradient-Based Optimizer algorithm	✔	✔	✖	✖	✖	✖	2024	Alghamdi, Ali S.^7^
Slime Mold Algorithm	✔	✔	✖	✖	✔	✖	2023	Chakraborty A. et al.^9^
Improved crow search algorithm	✔	✖	✖	✔	✖	✖	2021	Bakhshaei et al.^63^
Pelican Optimization Algorithm	✔	✖	✖	✔	✖	✖	2023	Nehmedo et al.^64^
Jellyfish Search Optimization Algorithm	✔	✔	✔	✖	✖	✖	2023	Datta et al.^65^
Hybrid Gray Wolf-Whale Optimization	✔	✔	✖	✔	✖	✖	2023	Datta et al.^66^
Chaotic Aquila Optimization	✖	✔	✖	✖	✖	✖	2023	Kujur et al.^67^
Slime Mold Algorithm	✔	✔	✖	✖	✔	✖	2024	Chakraborty et al.^68^
Slime Mold Algorithm	✔	✔	✖	✖	✔	✖	2024	El-Bayeh et al.^69^
DE Algorithm	✔	✔	✔	✔	✔	✔	Our article	

**Figure 2:**
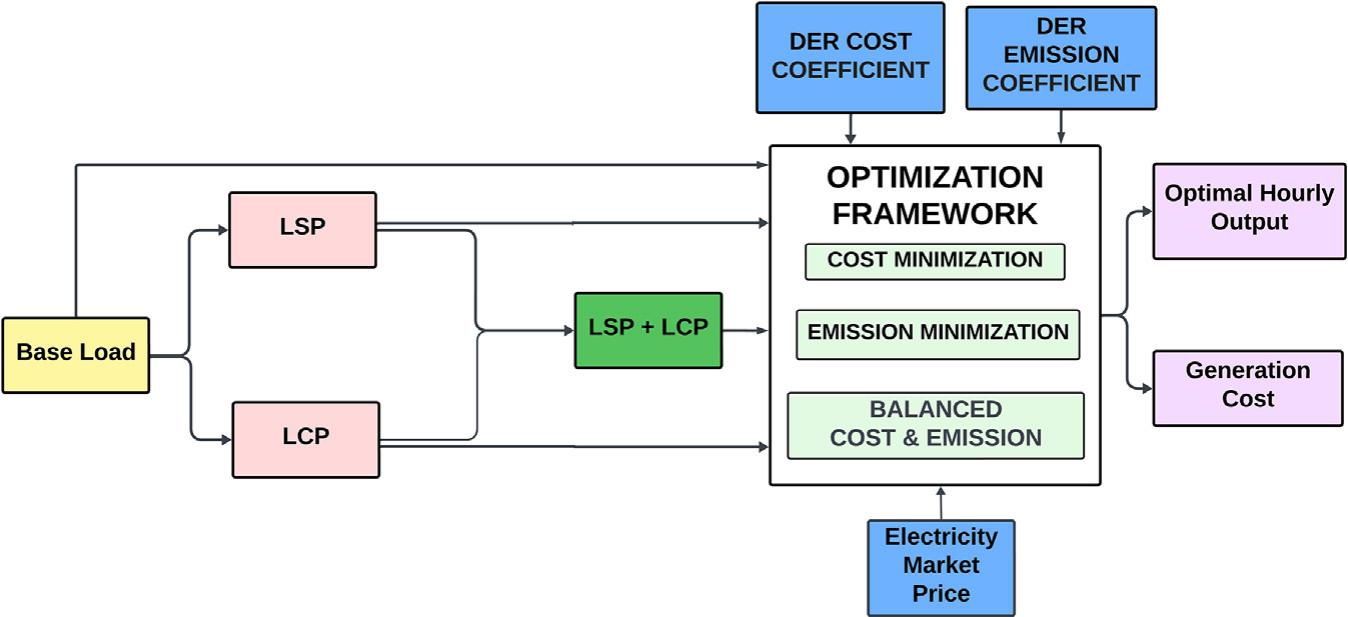
Flow chart of the work done in the article

The work done in this research is summarized in Figure 2, which includes the aspects discussed previously.

The proposed hybrid demand-side management (DSM) policy is designed to be flexible and scalable, making it well-suited to integrate emerging technologies such as advanced energy storage systems, vehicle-to-grid (V2G) capabilities, and more efficient renewable energy sources. By leveraging a modular framework and advanced optimization techniques such as the Differential Evolution (DE) algorithm, the system can accommodate technological advancements without requiring significant structural changes. For instance, the integration of energy storage technologies, such as next-generation batteries or grid-scale solutions, can be seamlessly incorporated into the existing optimization framework. These storage systems would further enhance the ability to balance supply and demand, store surplus renewable energy, and manage peak loads more effectively. Similarly, the proposal accounts for V2G capabilities by already incorporating smart PHEV charging strategies, which can be expanded to include bidirectional energy flows. This would allow PHEVs to act as mobile energy storage units, contributing to grid stability and increasing the utilization of renewable energy. Additionally, as renewable energy technologies become more efficient and widespread, the system’s modeling framework, including Hong’s 2m Point Estimation Method (PEM), ensures that uncertainty in renewable output is addressed dynamically. The optimization approach is designed to evolve with changing energy generation profiles, enabling the system to maintain relevance and effectiveness as renewable penetration increases. To ensure long-term relevance, the proposed policy is intentionally adaptable, allowing for iterative updates based on technological trends, regulatory changes, and consumer preferences. By continuously integrating advancements in energy systems and leveraging predictive analytics, the proposal can evolve alongside emerging technologies, maintaining its position as a robust and effective solution for smart grid optimization.

### Organization of the article

Section 2 explains the mathematical modeling for the fitness function of minimizing the generation cost, pollutants emitted, and balanced economic emission operation for the subject MG system. The modeling of DERs along with PHEV which includes their constraints are also explained in detail in this section along with the mathematical framework of LSP and LCP. Section 3 describes in detail about the framework of the differential evolution algorithm which is the proposed optimization tool. Section 4 implements the proposed strategy in a subject MG system and presents a detailed discussion of the results obtained with necessary figures and tables. Section 5 discusses the uncertainties in each case and the best performing case. Section 6 discusses the challenges and implications of the proposed research problem. Section 7 concludes the conclusion of the proposed research problem with a proper justification.

### Mathematical formulation

#### Formulation of fitness function

Due to the unpredictability and randomness of RES uncertainty in RES output has to be considered. The uncertainty in the expected value of the generation output is because of intermittence and uncertainty in the nature of the RES. Equations 1 and 2 are the mathematical equations for the calculation of uncertainty.

(Equation 1) $$P_{PV,un}^{t}=\Delta P_{PV,un}\times n_{1}+P_{PV,fc}^{t}\;\Delta P_{PV,un}=0.7\times \sqrt{P_{PV,fc}^{t}}$$

(Equation 2) $$P_{W,un}^{t}=\Delta P_{W,un}\times n_{2}+P_{W,fc}^{t}\;\Delta P_{W,un}=0.7\times \sqrt{P_{W,fc}^{t}}$$

Where Δ*P*_*PV*,*n*_ - standard deviation (SD) of the output of the PV. Δ*P*_*W*,*n*_-standard deviation (SD) of the output of the wind generation. $P_{PV,un}^{t}$ is considered as the uncertainty of the output of PV. $P_{PV,fc}^{t}$ is the day-ahead forecasting value of the output of PV. $P_{W,un}^{t}$ is the uncertainty of output of wind and *P* $P_{W,fc}^{t}$ is day ahead forecasting value of the output of wind. *n*_1_and *n*_2_ are the random distribution with SD of 0 and mean of 1.

#### Strategy to manage plug-in hybrid electric vehicle charging demand

PHEVs have low battery capacity. Due to that reason, they need quick access to charging stations. So, access charging stations are constructed not only in public areas but also in residential areas to prevent that problem. Electric Vehicle Plug-in hybrid electric vehicles' charging demand is stochastic and depends on many factors. The models utilized for the total demand of charging PHEVs at home and public stations are Weibull probability density function (W-PDF) and normal probability density function (PDF), respectively. The mean and standard deviation values of the PDFs are calculated based on the specifications given on PHEVs. In the following, three charging patterns demonstrate the probabilistic nature of PHEVs, Uncontrolled charging allows PHEVs to join the network and charge at any station without time-related constraints. The regulated charging approach uses the times of low demand, typically between 9 p.m. and 6 a.m., to charge PHEV batteries. The smart charging plan is coordinated to charge PHEVs at times of low cost and surplus energy in the infrastructure. The proposed regulated and intelligent charging method appropriately manages the higher demand for charging plug-in hybrid electric vehicles targeting peak load reduction. This is depicted in the following equation used by Chakraborty et al.^74^:

(Equation 3) $$\mathrm{Min}\left(\frac{\omega_{m}\left(t\right)}{\beta_{PHEV}\left(t\right)}\times \sigma_{PHEV}\left(t\right)\right)$$

An established coefficient *β*_PHEV_ represents the probability that a PHEV is located at a viable charging spot. The variable ω_*m*_(*t*) presents the hourly price of energy. The variable *σ*_PHEV_(*t*) represents the mean PHEV charging demand in kilowatts per hour.

For charging method during the controlled process:

(Equation 4) $$\sum_{t=1}^{T1}\beta_{PHEV}\left(t\right)+\sum_{t=21}^{T2}\beta_{PHEV}\left(t\right)=E_{Total}$$

For the method of smart charging:

(Equation 5) $$\sum_{t=1}^{T}\beta_{PHEV}\left(t\right)=E_{Total}$$

Here, the *β*_PHEV_ stays among *β*_PHEV,min_ and *β*_PHEV,max_

Values can be expressed as:

(Equation 6) $$\beta_{PHEV,\min}\leq \beta_{PHEV}\left(t\right)\leq \beta_{PHEV,\max}$$

*β*_PHEV,min_ and *β*_PHEV,max_ indicate the minimum & Maximum values of the PHEV demand (kW) hourly basis, respectively. “*E*_*Total*_” represents the total energy demand of the PHEVs (kWh). “*t*” indicates the index of the time.

#### Proposed load shifting policy (LSP)

**Figure 3:**
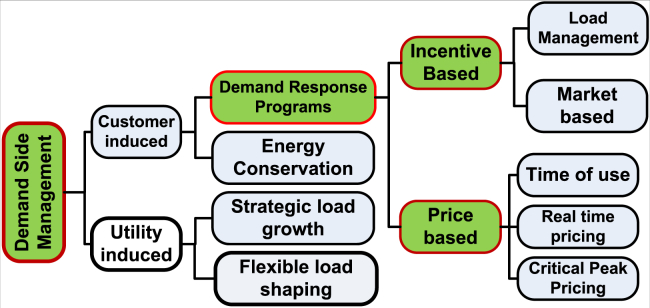
DSM Implementation Methods

This section provides an in-depth analysis related to the DSM technique and its mathematical models that are primarily used for optimization. The most availed strategy for load management from the six offered is load shifting. It does combine peak clipping and valley filling techniques. With the use of adjustable loads at the end, consumers create a way to facilitate the transfer of power demand. A reasonable adjustment in energy consumption may be obtained with the help of the load shifting method, which involves transferring controlled loads from peak to off-peak energy consumption. Figure 3 illustrates the various techniques employed for load shaping. Important components of load modeling using DSM are.

Step 1. Specify the varying demand across a time interval of T hours.
Step 2. Please provide the utility’s Time-of-Use.
*Step 3.* Adjust the involvement rate for Demand-Side Management when the shiftable loads are not clearly delineated.
*Step 4.* Identify non-shiftable loads and shiftable based on the percentage of engagement in DSM. For instance, if by x % DSM reveals that x % of demand at each time index is potentially shiftable, then remaining of (100-x) % is identified as non-shiftable load. The Load Profile Optimization is conducted with regard to the elastic load demand.
*Step 5.* Obtain the minimum and maximum values, along with the total, of the inelastic load demand. Special attention should be given to the need of optimising the elastic load.
Step 6. Minimize with Equation 21, by applying the optimization approach,
*Step7.* Thus, inserting the time index value of non-shiftable load into the optimal planned values of DSM for shiftable load, a modified load model was developed to be used the same as by Ahmad et al.^75^

The per cent of total load demand that determines DSM level is the amount of a controlled or an elastic load. Technically, DSM may be between 0% and 100%, depending on the elastic load. Expected load demand is that load demand that will result in the lowest operative cost. To get the expected load, the whole load needed is either assumed to be elastic or controlled. Despite its lack of realism, it is used to assess the resilience of a piece of work or strategy.

#### Hong’s 2m point estimation method for uncertainty modeling

There are several significant uncertainties in the MG operating problem that need to be assessed, and the right approach for addressing uncertainties has to be used. The optimal approach among the available options is determined by a number of crucial elements, including the level of precision needed and the body of knowledge regarding the behaviors of unknown parameters. This study introduces an PEM approach, based on Hong’s 2m PEM, which has been effectively employed to address the inherent uncertainty in the given scenario. The key advantages of this proposed PEM methodology include its swift implementation, improved accuracy in uncertainty modeling, and enhanced computational efficiency for complex power system problems. The core principle of PEM involves leveraging the solution set from the deterministic problem to derive statistical insights about the output variables, using only a limited number of estimates for the random input variables. Additionally, the wind power plant’s power output, as a function of wind speed, is modeled utilizing Weibull Probability Density Functions (PDFs) in accordance with the suggested Hong’s 2m PEM technique.

(Equation 7) $$\left(\mu_{p1},\mu_{p1},.....,p_{l,k},.....\mu_{pm}\right)$$

(Equation 8) $$p_{l,k}=\mu_{pl}+\zeta_{l,k}\theta_{pl}$$

(Equation 9) $$\zeta_{l,1}=\frac{\lambda_{l,3}}{2}+\sqrt{m+{\left(\frac{\lambda_{l,3}}{2}\right)}^{2}}$$

(Equation 10) $$\zeta_{l,2}=\frac{\lambda_{l,4}}{2}+\sqrt{m+{\left(\frac{\lambda_{l,4}}{2}\right)}^{2}}$$

(Equation 11) $$\omega_{l,1}=\frac{{\left(-1\right)}^{3-k}}{m}\times \frac{\zeta_{l,2}}{\zeta_{l,1}-\zeta_{l,2}}$$

(Equation 12) $$\omega_{l,2}=\frac{{\left(-1\right)}^{3-k}}{m}\times \frac{\zeta_{l,1}}{\zeta_{l,1}-\zeta_{l,2}}$$

(Equation 13) $$\lambda_{l,j}=\frac{M_{j}\left(pl\right)}{{\left(\theta_{pl}\right)}^{j}}$$

(Equation 14) $$M_{j}\left(pl\right)=\int_{-\infty }^{\infty }{\left(pl-\mu_{pl}\right)}^{j}f_{pl}d_{pl}$$

(Equation 15) $$\mu_{j}=X\left[Z^{j}\right]≅\sum_{l=1}^{m}\sum_{k=1}^{k}\omega_{l,k}{\left(Z\left(l,k\right)\right)}^{j}$$

(Equation 16) $$\sigma_{j}=\sqrt{X\left(Z^{1}\right)-{\left(X\left(Z^{2}\right)\right)}^{2}}$$

To further expand understanding of the proposed operational model of Hong’s 2m PEM operation, the general procedure of the PEM method may briefly be outlined as follows.

(1) Step 1: The value specified for the 1^st^ and 2^nd^ moments of the *k*^*th*^ output uncertain parameters is denoted as E(Z) = 0.
(2) Step 2: Choose the input constraints that are unclear, such as solar irradiance (k) or wind speed.
(3) Step 3: Uses of Equations 9, 10, 11, 12, 13, and 14 provide the values of the standard location and weight coefficients for the variable.
(4) Step 4: Using the greatest weight coefficients of k, find the 2m point.
(5) Step 5: For every concentration, solve the deterministic operational model technique.
(6) Step 6: Revise the output variables' raw moments
(7) Step 7: With Equations 15 and 16 as bases, calculate the statistical information of output parameters after repeating steps 2 to 6 until consideration for all concentration values of total input uncertain parameters is considered.

#### Differential evolution algorithm

**Figure 4:**
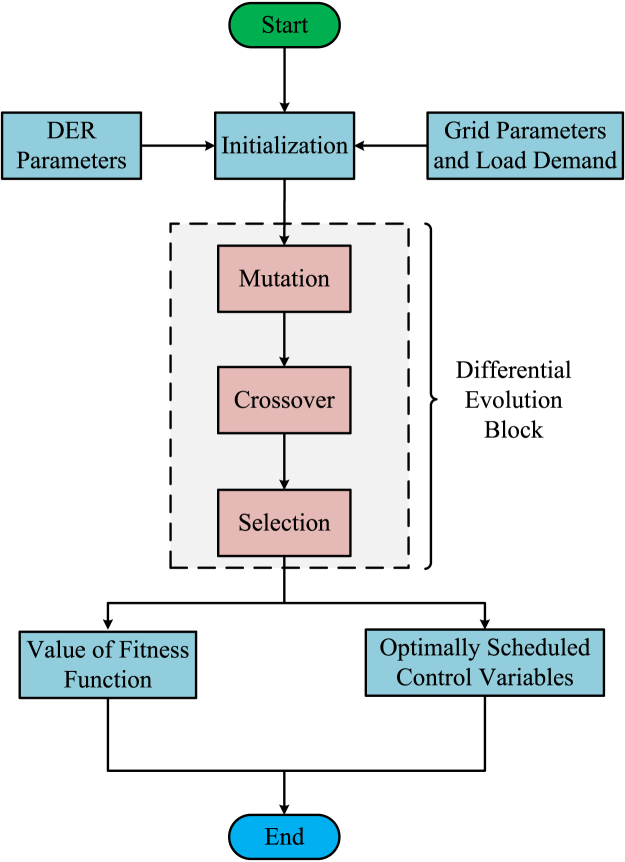
Implementation flowchart for Differential Evolution

In 1997, Storn and Price co-invented the DE Algorithm. Differential Evolution is one type of population-based evolutionary algorithm that could handle objective functions to be nonlinear and non-differentiable and may have multiple modes. From each parent individual of the population, a new offspring, called a trial vector is generated. The population is updated iteratively through the operations of selection, crossover, and mutation in a similar way given in Dey et. Al.^76^ To improve the new objective value for each candidate and to add it to the population that is considered for the next iteration of the algorithm. Otherwise, the newly computed objective value is ignored. Iteration of the procedure continues until a predetermined termination condition is reached. There is a natural advantage of DE in having a very few numbers of control parameters, which the user needs to alter. The set of parameters required for selection are population size NP, which has to be at least 4, mutation factor, known as differential scaling factor or weight, F, which lies in the range 0–2, and the probability of crossover, also called crossover control constraint, CR, lying in between 0 and 1. In the typical DE method, control restrictions were kept at a permanent value for the entire period of optimization. The population size affects the exploration capability of the algorithm. While the problem has many dimensions, the population size should be big enough to enable highly dimensional design space exploration for the algorithm. Figure 4 presents a succinct summary of the use of Differential Evolution (DE) in addressing the microgrid energy management problem.

The initial population arrangement is determined by randomly generating individuals inside the perimeter constraints.

(Equation 17) $$M_{xy}^{0}={M_{y}}^{\min}+\delta \times \left({M_{y}}^{\min}-{M_{y}}^{\max}\right)$$

The variable "δ" provides consistent values throughout the range of 0–1. Fitness function is computed for each individual. The mathematical representation of the "Mutation" process as stipulated in Equation 18 respectively.

(Equation 18) $$M_{x}^{/k}=0.5\times {\vec{g}}_{x}+0.5\times {\vec{L}}_{x}$$

Let $M_{x}^{/k}$ represent the newly generated *x*^*th*^ population set as a result of a mutation operation at the *k*^*th*^ iteration. The global and local donor vectors, ${\vec{g}}_{x}^{k}$ and ${\vec{L}}_{x}^{k}$, respectively. Where *F ∈ [0,2]* is a scaling factor parameter. The generation of a donor vector during the mutation stage is followed by the execution of crossover to enhance the potential uniqueness of the population. It may be shown using Equation 19. After mutation the modified and updated criterion is represented in Equation 20.

(Equation 19) $$M_{xy}^{//k}=\left\{ \begin{array}{c} M_{xy}^{/k}\;if\delta <CR\\ M_{xy}^{k}\;Otherwise \end{array}\right.$$

(Equation 20) $$M_{x}^{k+1}=\left\{ \begin{array}{c} M_{x}^{//k}\;if\;f\left(M_{x}^{//k}\right)\leq f\left(M_{x}^{k}\right)\\ M_{x}^{k}\;Otherwise \end{array}\right.$$

## Results and discussion

### Test system

**Figure 5:**
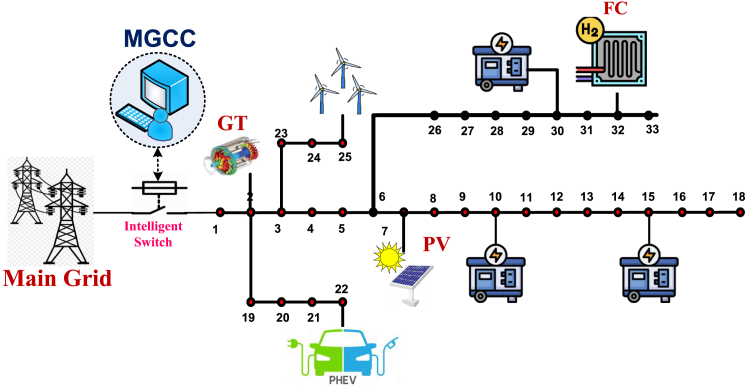
Modified microgrid system layout diagram

**Figure 6:**
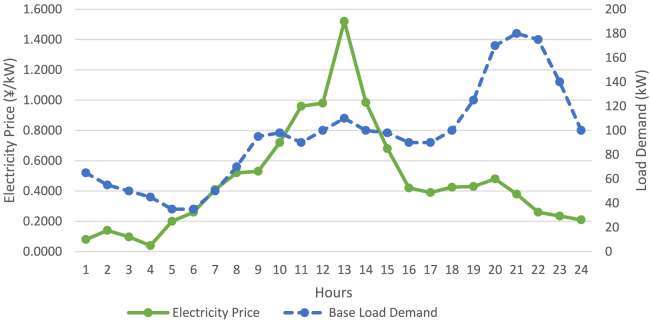
Base load demand and Electricity market price

**Figure 7:**
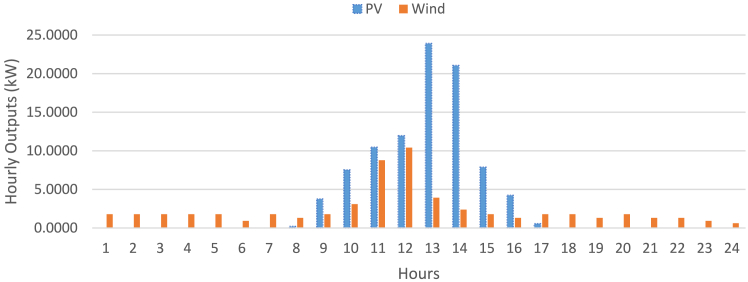
Day ahead forecasted output of renewable energy sources

A microgrid test system well connected with an LV grid, as represented by Figure 5, is considered using three traditional fuel-powered generating units, PV, Wind, GT, and FC. Table 2 presents the coefficients on the operational cost, coefficients of emission, and the upper and lower operational bounds of the DERs used by Singh et al.^77^ Table 3 presents price elasticity during peak, off-peak, and valley periods, respectively as Dey et. Al.^78^ The detail about PHEV is expressed in Table 4. Dynamic hourly power demands of the Microgrid System and the power market price based on TOU-based have been illustrated in Figure 6. Concerning the estimated parameters of RES, the results based on the uncertainty mentioned were computed the same as Singh et al.^77^ Where relevant, similar parameters were used to rank all objective functions for various circumstances. To source power from RES in a poor weather condition, out of total load demand only 2% was supplied by the RES. Their expenses were thus overlooked. The RES values used during the research work, considering uncertainty, are depicted in Figure 7. To promote transparency and reproducibility, the supporting information file will provide a comprehensive explanation of the data and models used in this study. The datasets, which were sourced from several referenced articles, have been appropriately cited within the article. These sources form the foundation for the load demand restructuring, DER scheduling, and PHEV smart charging strategies. Additionally, the data encompasses key parameters such as energy pricing, load patterns, and customer incentive structures, all of which were carefully selected to ensure the robustness of the analysis. The supporting information will also include detailed descriptions of the model implementation, particularly the application of the Differential Evolution (DE) algorithm. This will cover algorithmic parameters, such as population size, mutation rates, and stopping criteria, to offer full transparency regarding how the optimization was performed. By clearly outlining the data sources and model configurations, the supporting information will allow other researchers to replicate and build upon this work with confidence. Additionally, assumptions made throughout the analysis, such as customer participation rates and incentive effectiveness, will be discussed to provide a complete picture of the methodology.

**Table 2:** Description of DER scalars

Type	Parameters	D1	D2	D3	GT	FC	Grid
*dc*(¥/kW)	*Operational cost*	0.000116	0.000116	0.00006618	0.00008921	0.00002693	–
*om*(¥/kWh)		0.088	0.088	0.09	0.0648	0.0293	–
*fc*(¥/kWh)		0.396	0.396	0.396	0.695	0.206	–
CO_2_ (g/kW)	Emission coefficients	649	649	680	724	489	724
CO_2_ Treatment Cost(¥/kg)		0.21	0.21	0.21	0.21	0.21	0.21
Min.(kW)	Min-Max	0	0	0	6	3	−30
Max.(kW)		30	30	60	30	30	30

**Table 3:** Price Elasticity Matrix for incentive-based load curtailing policy

	Peak	Off Peak	Valley
Off Peak	0.02	−0.1	0.01
Valley	0.01	0.01	−0.1
Peak	−0.1	0.016	0.012

**Table 4:** Description of PHEV scalars

Parameters	Numerical values
Arrival time	09:00 HRS
Departure time	17:00 HRS
Arrival SOC	30%
Departure SOC	70%
Battery Capacity	50 kWh
Charging/Discharging power	±6 kW
Charging/Discharging rate	Same as Grid Price

### Descriptive analysis of results obtained

The work done in this article can be divided into stages with an aim to attain a balanced compromised magnitude of minimum power production price (PPP) with a lesser number of pollutants released in the atmosphere from the fossil-fuelled DERs. Five cases are studied based on the modified and restructured load demand and smart charging strategy of the PHEV. DE algorithm is used to percolate emission dispatch, economic dispatch, and balanced economic emission dispatch for all the five cases which are as follows.

Case 1: Base load demand
Case 2: Load demand modified as per load shifting policy of demand side management
Case 3: Load demand modified as per load curtailing policy of demand side management
Case 4: Load demand modified as per combined load shifting/curtailing policy
Case 5: Load demand modified as per combined load shifting/curtailing policy with smart PHEV charging, in short Case 5 is Case 4 with smart charging of PHEV. The stagewise explanation of the work done is explained in a descriptive manner with supporting figures and tables hereafter.

#### First stage: Restructuring the base load demand according to different cases

In this stage the base load demand is modified or restructured following the load shifting policy (Case 2), load curtailing policy (Case 3), and hybrid load shifting/curtailing policy (Case 4). It was considered that 40% of the customers participated to shift and curtail their loads. Put differently, it may also be said that 40% of MG system users committed to taking part in LSP. It should be mentioned that, in the end, the LSP has no effect on altering the total load demand, as well as the system’s average load, remains unchanged. It raises the load factor by lowering the peak load.

**Figure 8:**
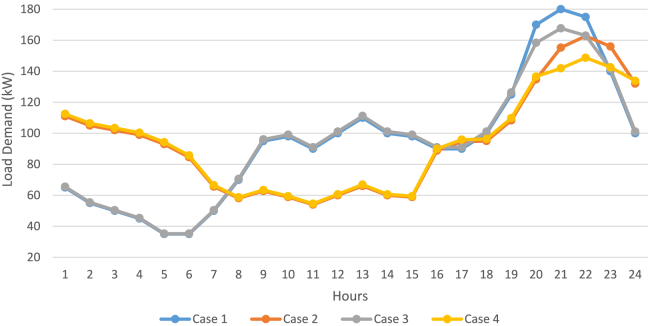
Modified load demand following load shifting, curtailing and combined load shifting/curtailing policy

In Case 3, the customer-friendly load curtailment policy (LCP) promises to reward users for reducing their loads during peak hours. Given that 40% of the MG system’s households consented to engage in LCP, LCP is being implemented in this instance. An extensive iterative technique was used to determine the ideal incentive value of 0.2¥, which resulted in the reduction of 18kW loads during peak hours. For the same, the clients received a 7¥ bonus. In Case 4, an innovative concept is applied to implement LCP on the load demand restructured in Case 2 post implementation of the LSP. Since, this case comprises of both shifting and curtailment of loads, this is named as hybrid load shifting curtailment policy (HLSCP). When 0.2¥ of ideal incentive value was considered for this case the amount of load curtailed was near to 17.5kW with 8¥ reward for the customer. Figure 8 shows the modified load demand post implementation of LSP, LCP, and HLSCP on the base load demand (Case 1). The lowest decrement of peak load can be seen in Case 5. Table 5 demonstrates the advantages of the load demand restructuring for the different cases. The peak load as mentioned above decreased from 180kW during Case 1 to 149kW in Case 5. The load factor increased from 0.52 to 0.63 during HLSCP and 18kWh energy was saved during Case 4 which also rewarded the consumers with 8¥ incentive.

**Table 5:** Economic evaluation and system performance for restructured load demand

Evaluation Parameters	Case 1	Case 2	Case 3	Case 4	Case 5
total load (kw)	2266.0000	2265,9990	2248.0122	2248.5115	2248.5115
energy saved (kWh)	0.0000	0.0000	17.9878	17.4885	17.4885
peak load (kW)	180.0000	162.6883	167.6721	148.7191	148.7191
load factor	0.5245	0.5804	0.5586	0.6300	0.6300
fuel+grid cost (¥)	707.0000	683.9826	688.5235	674.1062	668.3279
incentive cost (¥)	0.0000	0.0000	7.1913	8.1382	8.1382
Total operating cost (¥)	707.0000	683.9826	695.7148	682.2444	676.4661

#### Second stage: Optimal scheduling of distributed energy resources to minimize the generation cost of the microgrid system using differential evolution for cases 1 to 5

**Figure 9:**
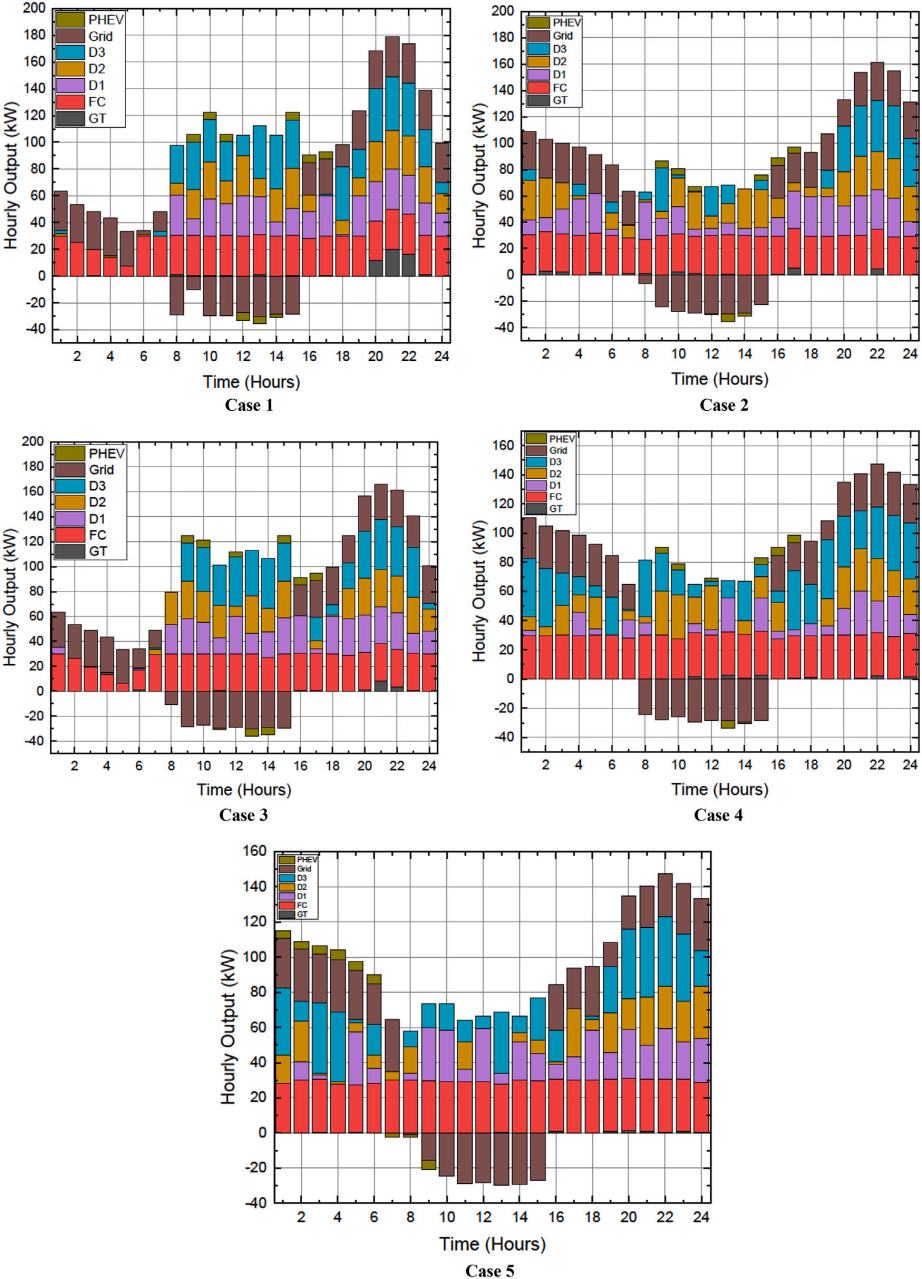
Energy Transactions under the presence of DE for Cases 1 to 5

In this stage, the generation expenses which comprises of the fuel cost and grid energy exchange cost is minimized using DE algorithm. It can be seen from Table 4 that the total operating cost to be borne by the MG operator was 707¥ in Case 1 (base load demand) which gradually reduced to 682¥ during Case 4 and then 676¥ during Case 5. It is also to be noted that the total operating cost during Cases 4 and 5 comprised of the 8¥ incentives to be awarded to the customers. The 6¥ reduction in the total operating cost of Case 5 compared to Case 4 is due to the smart charging strategy of the PHEV. According to the smart charging strategy, the PHEV exchange power with the MG during the hours when the electricity price is less. It can be seen in Figure 5 that the electricity price was less during first 9 h. Figure 9 depicts the energy transactions under the presence of DE. In all the cases it can be seen that the grid buys back power from the MG system when the electricity price is highest. This is a crucial factor in the generation cost minimization of an MG system. The PHEV can be seen to be charging and discharging during its default 08:00HRS to 17:00HRS for cases 1 to 4, except in Case 5 when the smart charging strategy was implemented the PHEV utilized 0100HRS to 0900HRS to meet its demand thereby further reducing the total operating cost of the MG system.

**Figure 10:**
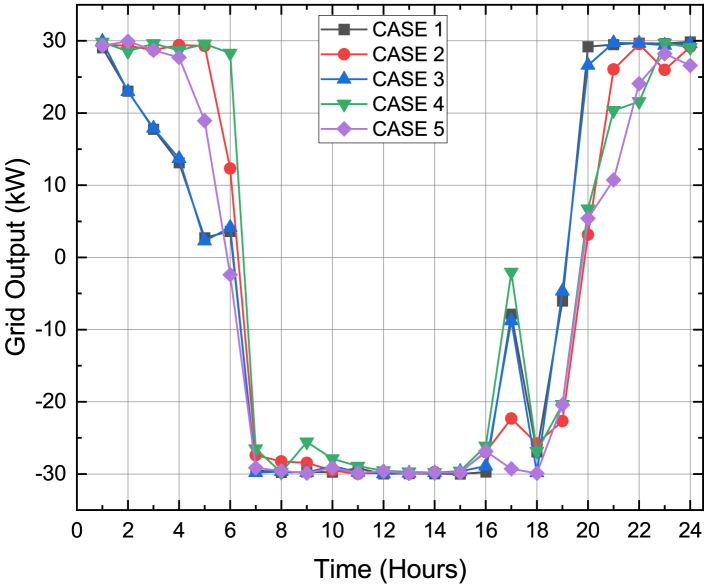
Response of grid for minimum generation costs for cases studied

**Figure 11:**
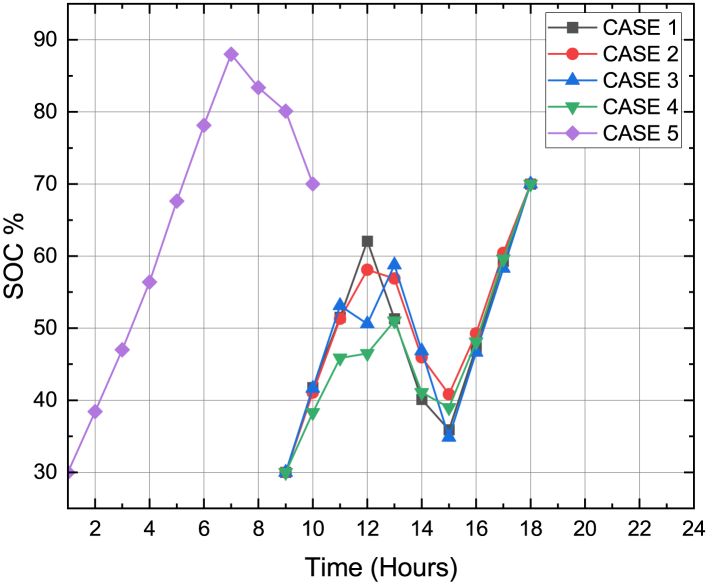
SOC of PHEV w.r.t hours recorded when generation cost was minimized using DE for Cases 1 to 5

**Figure 12:**
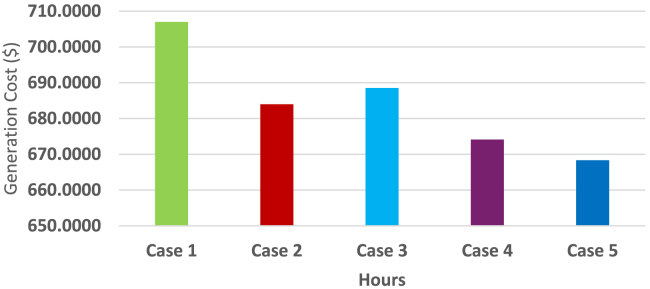
Minimum generation costs for five cases as obtained using DE

Figure 10 shows the contribution of the grid in energy transactions. The SOC constraint of the PHEV was maintained between 30% and 70% between its arrival and departure time for all the cases and this was depicted in Figure 11. Figure 12 sums up the 2^nd^ stage of the work done in this article. It shows that the minimum generation cost was obtained when the load demand was restructured as per innovative HLSCP with PHEV utilizing the smart charging strategy.

#### Third stage: Optimal scheduling of DERs to minimize the toxic emissions using DE for Cases 1 to 5

In this stage, DE is implemented to optimally schedule the DERs in a way that minimizes the carbon footprints emitted by the fossil fuels and grid. This is done by minimizing Equation 2 for all the cases. The minimum value of pollutants emitted was 1241kg for cases 1 and 2 and 1227kg for the rest of the three cases. This means that for the carbon footprint is primarily dependent on the total day ahead energy consumption of the MG system. For the first 2 cases when energy consumption remained unaltered it was 1241kg. The subsequent three cases witnessed a curtailment of 18kW load which decreased the carbon footprint to 1227kg. This also means that smart charging has no impact on the decrement of the carbon footprint of the system.

#### Fourth stage: Optimal scheduling of distributed energy resources to obtain balanced economic emission dispatch using differential evolution for cases 1 to 5

**Figure 13:**
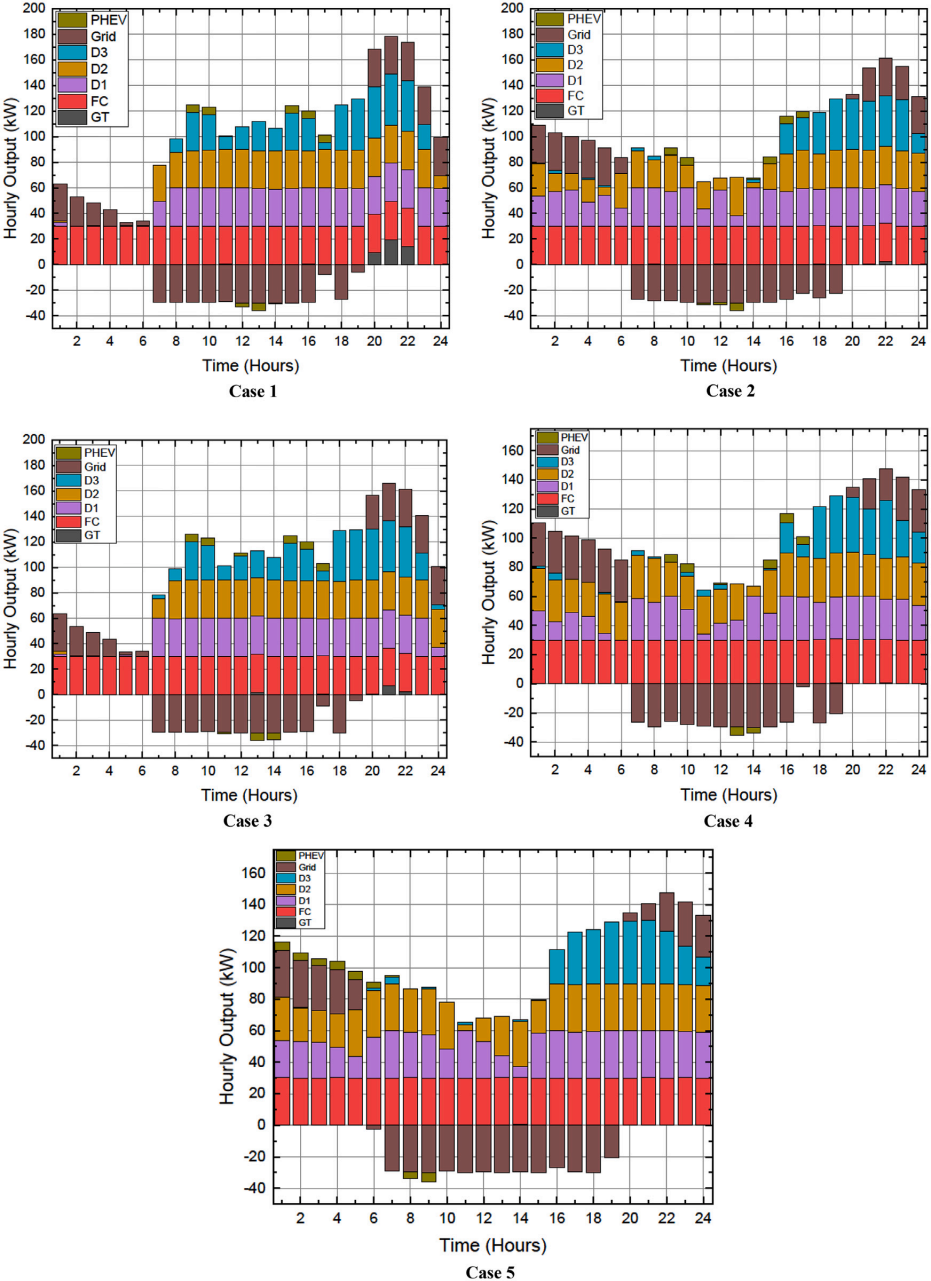
Hourly out of DERs when balanced economic emission dispatch was obtained using DE for Cases 1 to 5

Environment constrained economic dispatch (ECED) aims to attain a trade-off between the minimum value of total operating cost and carbon emissions giving equal weightage to both. In the 4^th^ stage of this work, ECED was evaluated for all the cases mentioned above. The balanced minimum cost-emission pair for various cases is displayed in Table 6. The best and minimum cost with compromised carbon emission is seen for Case 5 (685¥, 1246kg) wherein the total operating cost was also the least as reported in Table 5, 2^nd^ stage. Figure 13 presents the hourly generation of Distributed Energy Resources (DERs) across all scenarios, where the ECED was determined by minimizing Equation 4 with μ = 0.5.

**Table 6:** Values of cost (¥) and emission (kg) for different objective functions during Cases 1 to 5

Obj.Fn	Case 1		Case 2		Case 3		Case 4		Case 5	
	Cost	Emission	Cost	Emission	Cost	Emission	Cost	Emission	Cost	Emission
ECD	707	1301	684	1291	688	1290	674	1282	668	1283
EMD	872	1241	858	1241	858	1228	848	1227	871	1227
ECED	720	1267	690	1262	709	1254	694	1253	685	1246

**Figure 14:**
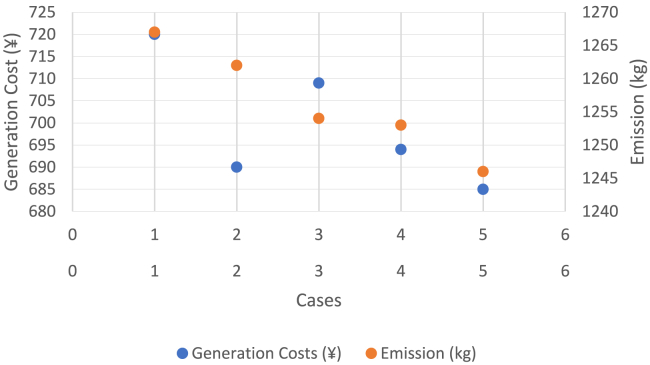
Scattered diagram for balanced cost-emission pair for 5 cases

**Figure 15:**
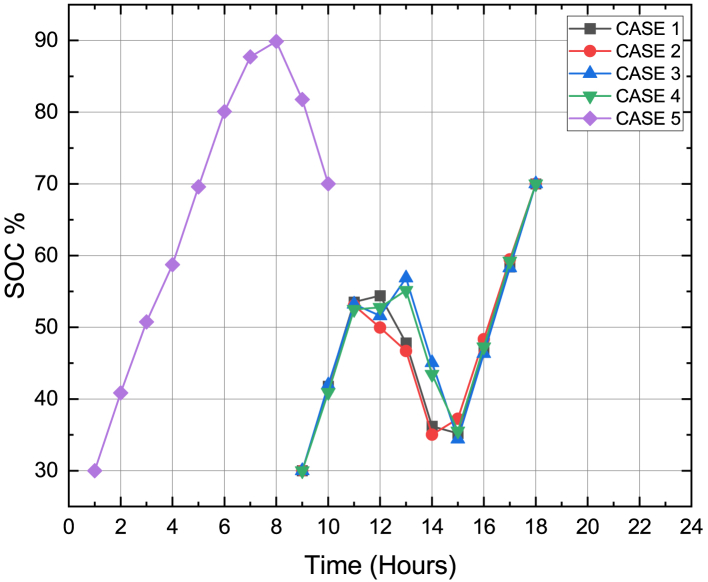
SOC of PHEV w.r.t hours recorded when balanced economic emission dispatch was obtained using DE for Cases 1 to 5

Figure 14 gives a distinct pictorial representation of Table 6. The best and minimal compromised of total operating costs and carbon emission is clearly the one obtained during Case 5 which involved proposed HLSCP with smart charging of PHEV. Figure 15 shows that the SOC level of PHEV was maintained between 30% and 70% during its availability in the charging station.

#### Fifth stage: Comparison of results obtained with recent literature and analyzing the quality of results obtained using the differential evolution algorithm

Table 7 later in discussion represents a comparison between the results obtained in this work with that available from recently published reputed articles. The minimum generation cost reported was 783¥ using an MGWOSCACSA optimizer as reported by Singh et al.,^77^ 743¥ using a hybrid CSAOA reported by Dey et al.,^79^ whereas DE outperformed the reported values with 707¥ in this work. Similarly, minimum value of carbon emissions was reported 1316kgs Singh et al.^77^^,^ Dey et al.,^79^ which was further lowered to 1241kgs in this work. The balanced cost-emission pair obtained using DE in this work was (720¥, 1267kgs) without DSM, (690¥, 1262kgs) with DSM and (685¥, 1246kgs) with proposed HLSCP with smart PHEV charging. These values clearly outperformed all of the reported values in recent literature and can be seen in Table 7.

**Table 7:** Comparative analysis of results obtained with recent literatures

Objective	Values in reported literature	This work
Generation cost minimization (¥)	783^77^; 743^79^	707
Carbon emission minimization (kg)	1316^77^^,^^79^	1241
ECED without DSM (¥, kg)	(814, 1341)^77^; (760, 1268)^79^	(720, 1267)
ECED with DSM (¥, kg)	(735, 1272)^79^	(690, 1262)
ECED with HLSCP and smart PHEV charging		(685, 1246)

**Figure 16:**
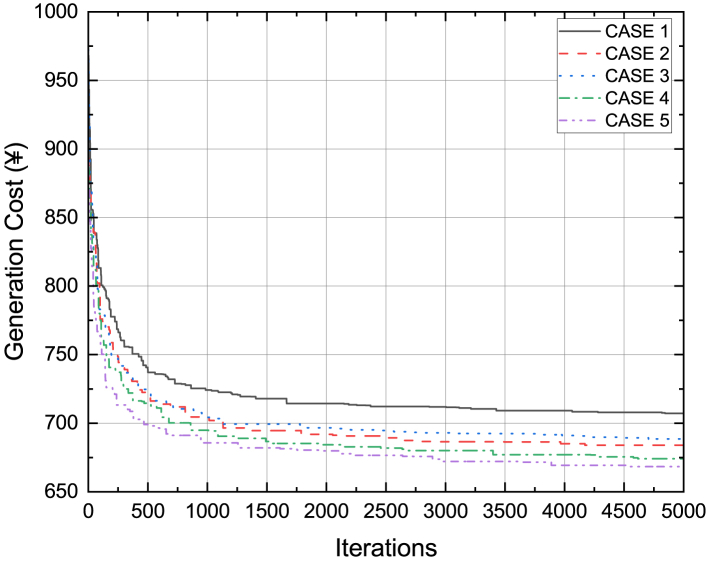
Cost convergence curves when generation cost was minimized using DE

**Figure 17:**
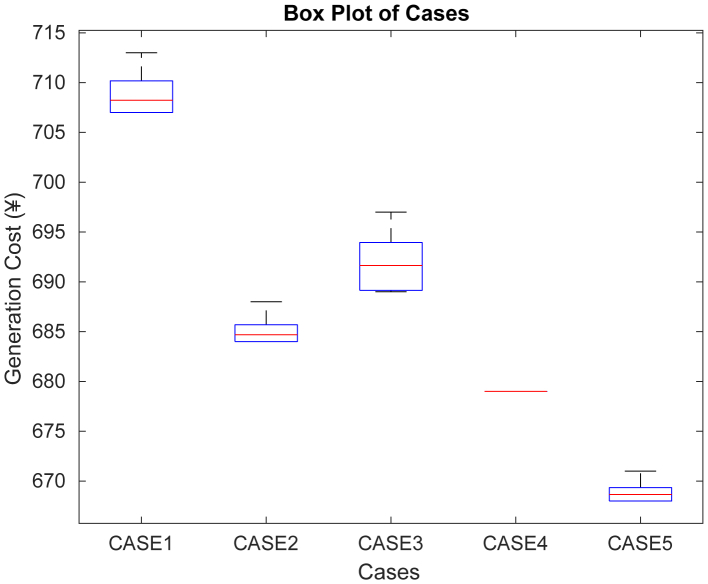
Boxplot from the statistical data obtained after 30 trials of DE for all the case

In Cases 1 through 5, DE was executed for 30 independent trials in order to minimize the generating cost. The results were recorded together with the amount of time that had passed before the halting threshold was reached. The lowest, highest, and average generation cost values found with DE are shown in Table 8. The robustness of the suggested DE method in the shortest amount of time is indicated by the minimal standard deviation value and high hits value. When cost was reduced using DE for Cases 1 through 5, the convergence curve features are displayed in Figure 16. The boxplot figure created using Table 8’s statistical data is displayed in Figure 17.

**Table 8:** Nonparametric statistical data for DE algorithm

Cases	Minimum (¥)	Maximum (¥)	Average (¥)	STD	Hits	Time (sec)
1	707	713	708.4	2.5811	23	4.3
2	684	688	684.6667	1.5162	25	3.99
3	689	697	691.1333	3.5982	22	4.23
4	674	679	687.6667	3.4575	26	3.55
5	668	671	668.5	1.1371	25	3.25

### Uncertainties in each case and the best performing case

In all five cases examined, various uncertainties emerge from factors such as demand fluctuations, the precision of smart charging algorithms, and consumer responses to incentive programs. Specifically, Case 5 introduces additional uncertainties tied to the reliability of price forecasts, electricity price fluctuations, and the responsiveness of the smart charging system. This strategy’s success hinges on accurate energy price predictions and effective management of energy exchanges. Furthermore, assumptions about customer participation rates (40% in this scenario) and responsiveness to incentives are critical. Any deviations, such as unforeseen price spikes or shifts in demand, could impact the scalability and reliability of this approach. Despite these challenges, Case 5 achieved significant improvements in system performance, including a reduction in peak load to 148.72kW (compared to 180kW in Case 1) and the highest load factor of 0.6300 among all cases. These results highlight the hybrid DSM approach’s capacity to optimize both grid sustainability and operational efficiency.

## Discussion

Scaling up the best-performing scenario (Case 5) to a larger system presents several practical challenges and considerations. One major obstacle is the reliance on high participation rates in demand-side management (DSM) programs, such as load shifting and curtailment. Achieving and maintaining a 40% participation rate may prove difficult over time, as sustaining customer engagement can be challenging. Additionally, incentive structures, such as the 8¥ rewards offered, may require frequent adjustments to remain effective, which could lead to higher administrative and operational expenses. From a technological perspective, the success of smart PHEV charging systems hinges on the smooth interaction between the grid and vehicles, necessitating a reliable and scalable infrastructure. While integrating smart charging into the energy mix can lead to cost savings and reduced emissions, it also introduces complications related to maintaining grid stability, particularly during unexpected demand spikes or volatile electricity prices. Furthermore, widespread implementation of this strategy would face regulatory complexities, as incentive models and energy trading frameworks must comply with local policies and market dynamics. The financial burden of providing incentives on a larger scale could also impact the overall cost-benefit equation. Nonetheless, despite these obstacles, the potential for significant reductions in both operational costs and emissions makes Case 5 an attractive option for continued research and development.

### Conclusion

In this research, a unique method was proposed to reorganize the predicted load demand of a microgrid (MG) system by combining curtailment and load-shifting strategies. The microgrid system’s G2V and V2G capabilities facilitated the charging and discharging of plug-in hybrid electric vehicles (PHEVs). Five stages demonstrating balanced economic emission dispatch, emission dispatch, and economic dispatch for five distinct load demand models, with and without considering LSP, LCP, or HLSCP, were carefully examined. The hybrid DSM approach addresses the critical challenge of balancing economic and environmental objectives in microgrid systems. By integrating load shifting, curtailment, and smart PHEV charging, this strategy delivered reductions in peak demand and emissions while increasing load factor and lowering generation costs, demonstrating its value for sustainable energy management. Additionally, it compensates clients by offering incentives for their participation in these strategies. Among the five load demand models, the HLSCP with smart PHEV charging provided a superior trade-off between minimal generating cost and emissions.

Numerical results revealed that while the total load demand remained relatively unchanged, the modified policies led to notable improvements in system performance. Energy savings were observed in Cases 3, 4, and 5, with 17.99 kWh saved in Case 3 (Load Curtailment Policy) and approximately 17.49 kWh saved in Cases 4 and 5 (Hybrid Load Shifting/Curtailing with Smart PHEV Charging). Case 2 (Load Shifting Policy) showed no energy savings, as it only shifted the load without reducing total demand. The peak load was significantly reduced in all modified cases, with Cases 4 and 5 achieving the lowest peak load of 148.72 kW, compared to 180 kW in the base case (Case 1). This reduction highlights the effectiveness of DSM strategies in improving load management. Furthermore, the load factor improved across all modified cases, with Cases 4 and 5 demonstrating the highest load factor of 0.6300, indicating more efficient and stable system operation. In terms of cost, fuel and grid expenses decreased in all modified cases, with Case 5 showing the lowest total operating cost of ¥676.47, compared to ¥707.00 in the base case. These findings underscore the potential of integrating smart load management policies, including PHEV charging strategies, to optimize both economic performance and emission reductions in renewable-integrated power systems. However, this study did not include an analysis of voltage profiles, a standard distribution system, or the integration of dedicated energy storage systems. These limitations provide opportunities for future research to build on and extend this work. Additionally, the increasing number of PHEVs, with uncertainties and variations in their state of charge (SOC) and arrival/departure times, can amplify the complexity of MG operations, offering another avenue for further investigation. Future work will also explore the integration of advanced optimization techniques, such as dynamic load forecasting, to further enhance system performance. As part of the future scope, we plan to incorporate cybersecurity frameworks and evaluate the computational overhead associated with security measures to ensure reliable, secure, and efficient communication. Addressing these concerns will enhance the practical applicability of the proposed policy for large-scale, real-time smart grid implementations.

### Limitations of the study

This research work offers significant advancements in demand-side management (DSM) strategies for microgrid (MG) systems, several limitations should be acknowledged. First, the study did not include a detailed analysis of voltage profiles, which is critical for evaluating the stability and reliability of MG operations. Additionally, the framework was applied to a specific low-voltage MG system, without considering standard distribution networks, limiting the generalizability of the findings. The absence of dedicated energy storage systems in the analysis also constrains the flexibility and efficiency enhancements that could have been achieved under varying load conditions. Furthermore, assumptions regarding consumer participation rates and responsiveness to incentive programs, such as the 40% compliance rate used in the study, may not fully capture real-world behavioral variability, introducing uncertainties in the scalability of the proposed hybrid load-shifting and curtailment policy (HLSCP). The reliance on modeled energy price predictions for the smart PHEV charging strategy adds another layer of uncertainty, as real-world price fluctuations and market dynamics were not accounted for. Lastly, while cybersecurity was identified as an area for future exploration, this study did not address the practical implementation challenges or computational overhead of secure communication frameworks in real-time smart grid environments. These limitations provide avenues for future research to refine the proposed methodologies and ensure their applicability under diverse and realistic conditions.

## Resource availability

### Lead contact

Further information and requests for resources and reagents should be directed to and will be fulfilled by the lead contact, Bishwajit Dey (bishwajit.dey@jaipur.manipal.edu).

### Materials Availability

This study did not generate new unique materials.

### Data and code availability

• *Data:* All data reported in this article will be shared by the lead contact upon request.
• *Code*: This article does not report original code.
• Any additional information required to reanalyze the data reported in this article is available from the lead contact upon request.

## Acknowledgments

This research is funded by European Union under the REFRESH—Research Excellence For Region Sustainability and High-Tech Industries Project via the Operational Programme Just Transition under Grant CZ.10.03.01/00/22_003/0000048; in part by the National Centre for Energy II and ExPEDite Project a Research and Innovation Action to Support the Implementation of the Climate Neutral and Smart Cities Mission Project TN02000025; and in part by ExPEDite through European Union’s Horizon Mission Programme under Grant 101139527. The authors would like to express their sincere gratitude to Lukas Prokop and Stanislav Misak for their exceptional supervision, project administration, and overall guidance throughout the course of this project. Their expertise and support were instrumental to its success.

## Author contributions

A. R. S., B. D., and S. M.: conceptualization, methodology, software, visualization, investigation, and writing-original draft preparation. R. S. K.: data curation, validation, supervision, resources, and writing - review and editing. M. B. and V. B.: project administration, supervision, resources, writing - review and editing. All authors assisted during article preparation.

## Declaration of interests

Authors declare that there is no competing interest.

## STAR★Methods

### Key resources table

**Table:** 

REAGENT or RESOURCE	SOURCE	IDENTIFIER
**Deposited data**		

PV	Day ahead forecasted output of renewable energy sources	Figure 7
WT	Day ahead forecasted output of renewable energy sources	Figure 7
PHEV	Values	Table 4
OPERATIONAL COST	Values	Table 2
Price Elasticity Matrix	Values	Table 3
Modified Load demand	Values	Figure 8
DataSet for grid data could be found upon request from lead contact		

**Software and algorithms**		

MatlabR2022a	MathWorks.Inc	https://ww2.mathworks.cn/?s_tid=gn_logo

### Experimental model and study participant details

This study does not use experimental models.

### Method details

#### Restructuring of load demand according to load shifting and curtailing policies

##### Load shifting policy

The load shifting technique is the optimal financial approach that represents shifting of the flexible loads to the time when the utility charges lesser prices Misra et al.^80^ DSM implementation of their corresponding strategies can benefit from multiple angles in an electricity system. It saves costs, enhances load factor, and manages peak demand without impacting the overall load demand.

(Equation 21) $$Minimize\sum_{t=1}^{T}\left[c_{C}^{t}*\left(elst_{load}\;at\;t^{th}\;hour+enelst_{load}\;at\;t^{th}\;hour\right)\right]$$

where,

$$0\leq elst_{load}\;at\;t^{th}\;hour\leq maxmium\;elastic\;load$$

$$\sum_{t=1}^{T}P_{load}^{t}=\sum_{t=1}^{T}\left(elst_{load}\;at\;t^{th}\;hour+enelst_{load}\;at\;t^{th}\;hour\right)$$

##### Load curtailing policy

It is the utility corporations, business entities serving load, or local grid operators that decide demand response schemes. Incentives are offered to customers for reducing their loads. These could be provided through or separately from their retail energy cost. The retail price of electricity will either be fixed or vary depending on time, depending on the average cost. The grid operator demands and requests load reductions when they perceive a reliability risk or when prices become too high. Mostly the DR systems have a process for calculating an end-user’s baseline level of energy use, so end-users can vary accurately estimate and confirm the customer's level of response to changes in load. In some cases, customers would incur penalties if they enrolled in DRPs but did not respond to events or fell short of their contractual commitments. For that, the concept of elasticity demand and pricing is used to work out the economic model for the load.^3^

(Equation 22) $$L_{bs}^{DR}\left(t\right)=\eta L_{bs}^{0}\left(t\right)\left[1+E\left(t,t\right)\left(\frac{C_{g}\left(t\right)-C_{g_{0}}\left(t\right)+I\left(t\right)}{C_{g_{0}}\left(t\right)}\right)\right]+\sum_{h\neq 1,h\neq t}^{24}E\left(t,h\right)\left(\frac{C_{g}\left(h\right)-C_{g_{0}}\left(h\right)+I\left(h\right)}{C_{g_{0}}\left(h\right)}\right)$$

At the interval of time *t* in Equation 22, $L_{bs}^{0}\left(t\right)$ represents the starting demand value at bus *bs*. The spot price of electricity at time interval *t* is denoted by *C*_*g*_*(t),* whereas the original price of electricity is represented by *C*_*g*_*0(t).* The current electricity spot price allows consumers to adjust their demands. The incentive value denoted as I(t), is the compensation granted to customers participating in the program of demand response (DR) when they reduce or shift their load during hour *t*. The self-elasticity is represented by *E(t, t),* whereas the cross-elasticity is marked as *E(t, h).* The mathematical representation of elasticity is represented in Equation 23 respectively.

(Equation 23) $$E=\frac{C_{g_{0}}}{L_{0}}.\frac{\partial L}{\partial C_{g}}$$

The demand can respond to a changing electricity price in any of these few ways. Some loads are light and cannot be switched but can only be turned on or off. These loads, for instance, can't be switched from one time to the next. Hence, these demands are sensitive only once, and this sensitivity is represented by self-elasticity, the value of which is continually negative. The impact of DR programme effects is an implementation of an effect in a reduction of load, as shown in Equation 24.

(Equation 24) $$\Delta L_{bs}^{DR}\left(t\right)=\eta L_{bs}^{0}\left(t\right)-L_{bs}^{DR}\left(t\right)$$

$\Delta L_{bs}^{DR}\left(t\right)$ is a representation of the decrease in the load on bus bs at the interval of time *t* that occurred as a direct consequence of the DR program. At the interval of time *t,* the total load at bus *bs* is represented by the symbol *L*_*bs*_*,* and it may be stated in the manner that is described in the Equation 25.

(Equation 25) $$L_{bs}=\left(1-\eta \right)L_{bs}^{0}\left(t\right)+L_{bs}^{DR}\left(t\right)$$

##### Minimization of cost, emission and weighted economic emission dispatch based on different load models

The cost function of the generation for a grid-connected microgrid system are stated in Equation 26.

(Equation 26) $$GC=\sum_{t}^{24}\sum_{k=1}^{n}\left(C_{k}\times P_{k,t}+t_{Grid,t}\times P_{Grid,t}\right)$$

Where *P*_*k*_ is the output power of the *k*^*th*^ unit and *C*_*k*_ denotes the cost coefficient of the *k*^*th*^ unit. The electricity price offered by the utility is expressed as *t*_*grid*_. The total generation cost is calculated for ′*n*′ number of generating units and it is considered for 24 hours of operation.

$$C_{k}=C_{fc}+C_{om}+C_{dc}$$

*fc*: Fuel Expenses.

*om*: Operational and Maintenance Expenses.

*dc*: Expense of Depreciation.

The following equation, which represents various sources emitting carbon dioxide into the atmosphere using conventional fuel sources by the end of the day, gives the total quantity: Equation 27,

(Equation 27) $$GE=\sum_{t}^{24}\sum_{k=1}^{n}\left(CO_{2k}\times P_{k,t}+CO_{2Grid}\times P_{Grid,t}\right)$$

Where, *CO*_*2*_*:* Emission coefficient of carbon dioxide.

*GE:* Total carbon dioxide emissions.

Each amount of carbon dioxide produced by fossil-fuel-based generating power plants is penalized by some amount. This somewhat reduces the release of environmental pollutants and increases the entire cost of producing energy by the system. It is given by Equation 28.

(Equation 28) $$CEED_{ppf}=\sum_{t}^{24}\sum_{k=1}^{n}\left(\begin{array}{c} C_{k}\times P_{k,t}+t_{Grid,t}\times P_{Grid,t}+\\ \;ppf_{k}\times CO_{2k}\times P_{k,t}+ppf_{grid}\times CO_{2Grid}\times P_{Grid,t} \end{array}\right)$$

Where *CEED*_*ppf*_ is Combined Economic Emission Dispatch and *ppf* is Price penalty factor.

The two optimization approaches concerning the variables (26) and (27) were well balanced as taken from Singh et al.^77^ They made use of Equation 29 which combined two decision variables with varying objectives to enhance the answer.

(Equation 29) $$ECED=\mu \left[\frac{GC-GC_{\min}}{GC_{\max}-GC_{\min}}\right]+\left(1-\mu \right)\times \left[\frac{GE-GE_{\min}}{GE_{\max}-GE_{\min}}\right]$$

*GC*_*min*_ and *GE*_*min*_ are the optimal criteria generated by reducing Equations 26 and 27, correspondingly, as μ fluctuates between 0 and 1. The optimal parameter *GC*_*min*_ of Equation 26 and *GE*_*min*_ of Equation 27 attain maximum values of GC and GE, respectively.

Equations 30, 31, 32, and 33 describe operating limitations:

(Equation 30) $$\sum_{k=1}^{n}P_{k,t}+P_{Grid,t}=P_{Dt}$$

(Equation 31) $$\sum_{k=1}^{n}P_{k,t}+P_{RES,t}+P_{Grid,t}=P_{Dt}$$

(Equation 32) $$P_{k,\min}\leq P_{k}\leq P_{k,\max}$$

(Equation 33) $$P_{grid,\min}\leq P_{grid}\leq P_{grid,\max}$$

Where

*P*_*RES,t*_*:* Output power of the RES

*Dt*: The total Demand of the *t*^*th*^ hour.

### Quantification and statistical analysis

This study does not include statistical analysis or quantification.

## References

[1] Hai T., Alazzawi A.K., Mohamad Zain J., Oikawa H. (2023). A stochastic optimal scheduling of distributed energy resources with electric vehicles based on microgrid considering electricity price. Sustain. Energy Technol. Assessments.

[2] Fathy A. (2023). Bald eagle search optimizer-based energy management strategy for microgrid with renewable sources and electric vehicles. Appl. Energy.

[3] Chhualsingh T., Rao K.S., Rajesh P.S., Dey B. (2023). Effective demand response program addresing carbon constrained economic dispatch problem of a microgrid system. e-Prime-Advances in Electrical Engineering. Electron. Energy.

[4] Zheng Y., Xue X., Xi S., Xin W. (2024). Enhancing microgrid sustainability: Dynamic management of renewable resources and plug-in hybrid electric vehicles. J. Clean. Prod..

[5] Hassan A.M.S. (2024). Joint energy management with integration of renewable energy sources considering energy and reserve minimization. Elec. Power Syst. Res..

[6] Hai T., Zhou J., Alazzawi A.k., Muranaka T. (2023). Management of renewable-based multi-energy microgrids with energy storage and integrated electric vehicles considering uncertainties. J. Energy Storage.

[7] Alghamdi A.S. (2024). Microgrid energy management and scheduling utilizing energy storage and exchange incorporating improved gradient-based optimizer. J. Energy Storage.

[8] Wang Y., Wang B., Farjam H. (2024). Multi-objective scheduling and optimization for smart energy systems with energy hubs and microgrids. Eng. Sci. Technol. Int. J..

[9] Chakraborty A., Ray S. (2023). Operational cost minimization of a microgrid with optimum battery energy storage system and plug-in-hybrid electric vehicle charging impact using slime mould algorithm. Energy.

[10] Zhang T., Sobhani B. (2023). Optimal economic programming of an energy hub in the power system while taking into account the uncertainty of renewable resources, risk-taking and electric vehicles using a developed routing method. Energy.

[11] Hai T., Zhou J., Rezvani A., Le B.N., Oikawa H. (2023). Optimal energy management strategy for a renewable based microgrid with electric vehicles and demand response program. Elec. Power Syst. Res..

[12] Yang Z., Tian H., Min H., Yang F., Hu W., Su L., SaeidNahaei S. (2023). Optimal microgrid programming based on an energy storage system, price-based demand response, and distributed renewable energy resources. Util. Policy.

[13] Singh A., Kumar A., Chinmaya K.A., Maulik A. (2024). Optimal operation of an electricity-hydrogen DC microgrid with integrated demand response. Sustain. Energy Grids Netw..

[14] Ali Z.M., Al-Dhaifallah M., Alkhalaf S., Alaas Z., Jamali F. (2023). Optimal planning and design of a microgrid with integration of energy storage and electric vehicles considering cost savings and emissions reduction. J. Energy Storage.

[15] Hai T., Zhou J., khaki M. (2023). Optimal planning and design of integrated energy systems in a microgrid incorporating electric vehicles and fuel cell system. J. Power Sources.

[16] Bokopane L., Kusakana K., Vermaak H., Hohne A. (2024). Optimal power dispatching for a grid-connected electric vehicle charging station microgrid with renewable energy, battery storage and peer-to-peer energy sharing. J. Energy Storage.

[17] Armioun M., Nazar M.S., Shafie-khah M., Siano P. (2023). Optimal scheduling of CCHP-based resilient energy distribution system considering active microgrids' multi-carrier energy transactions. Appl. Energy.

[18] Annamalai T., Uthaya kumar G., Sivarajan S., Naga Malleswara Rao D. (2024). Optimized energy management for interconnected networked microgrids: A hybrid NEGCN-PFOA approach with demand response and marginal pricing. Energy.

[19] Roy N.B., Das D. (2023). Probabilistic optimal power dispatch in a droop controlled islanded microgrid in presence of renewable energy sources and PHEV load demand. Renew. Energy Focus.

[20] Singh A.R., Koteswara Raju D., Phani Raghav L., Seshu Kumar R. (2023). State-of-the-art review on energy management and control of networked microgrids. Sustain. Energy Technol. Assessments.

[21] Roy N.B., Das D. (2024). Stochastic power allocation of distributed tri-generation plants and energy storage units in a zero bus microgrid with electric vehicles and demand response. Renew. Sustain. Energy Rev..

[22] Alzahrani A., Rahman M.U., Hafeez G., Rukh G., Ali S., Murawwat S., Iftikhar F., Haider S.I., Khan M.I., Abed A.M., Abed A.M. (2023). A strategy for multi-objective energy optimization in smart grid considering renewable energy and batteries energy storage system. IEEE Access.

[23] Ali Z.M., Al-Dhaifallah M., Komikawa T. (2022). Optimal operation and scheduling of a multi-generation microgrid using grasshopper optimization algorithm with cost reduction. Soft Comput..

[24] Li M., Aksoy M., Samad S. (2024). Optimal energy management and scheduling of a microgrid with integrated electric vehicles and cost minimization. Soft Comput..

[25] Hai T., Aksoy M., Khaki M. (2024). Optimal planning and operation of power grid with electric vehicles considering cost reduction. Soft Comput..

[26] AL-Dhaifallah M., Ali Z.M., Alanazi M., Dadfar S., Fazaeli M.H. (2021). An efficient short-term energy management system for a microgrid with renewable power generation and electric vehicles. Neural Comput. Appl..

[27] Sudhakar A., Kumar B.M. (2024). EV Fleet Energy Management Strategy For Smart Microgrids Considering Multiple Objectives: Techno-Economic Perspective. Arabian J. Sci. Eng..

[28] Rajagopalan A., Nagarajan K., Bajaj M., Uthayakumar S., Prokop L., Blazek V. (2024). Multi-objective energy management in a renewable and EV-integrated microgrid using an iterative map-based self-adaptive crystal structure algorithm. Sci. Rep..

[29] Rahim S., Javaid N., Khan R.D., Nawaz N., Iqbal M. (2019). A convex optimization based decentralized real-time energy management model with the optimal integration of microgrid in smart grid. J. Clean. Prod..

[30] Tao H., Ahmed F.W., Abdalqadir kh ahmed H., Latifi M., Nakamura H., Li Y. (2021). Hybrid whale optimization and pattern search algorithm for day-ahead operation of a microgrid in the presence of electric vehicles and renewable energies. J. Clean. Prod..

[31] Lu X., Zhou K., Yang S. (2017). Multi-objective optimal dispatch of microgrid containing electric vehicles. J. Clean. Prod..

[32] Lu X., Zhou K., Yang S., Liu H. (2018). Multi-objective optimal load dispatch of microgrid with stochastic access of electric vehicles. J. Clean. Prod..

[33] Seyyedeh Barhagh S., Abapour M., Mohammadi-Ivatloo B. (2020). Optimal scheduling of electric vehicles and photovoltaic systems in residential complexes under real-time pricing mechanism. J. Clean. Prod..

[34] Sabyasachi S., Singh A.R., Godse R., Jaiswal S., Bajaj M., Srivastava I., Blazek V., Prokop L., Misak S. (2024). Reimagining E-mobility: A holistic business model for the electric vehicle charging ecosystem. Alex. Eng. J..

[35] Kumar B.A., Jyothi B., Singh A.R., Bajaj M., Rathore R.S., Tuka M.B. (2024). Hybrid genetic algorithm-simulated annealing based electric vehicle charging station placement for optimizing distribution network resilience. Sci. Rep..

[36] Nagarajan K., Rajagopalan A., Bajaj M., Sitharthan R., Dost Mohammadi S.A., Blazek V. (2024). Optimizing dynamic economic dispatch through an enhanced Cheetah-inspired algorithm for integrated renewable energy and demand-side management. Sci. Rep..

[37] Kumar B.A., Jyothi B., Singh A.R., Bajaj M., Rathore R.S., Berhanu M. (2024). A novel strategy towards efficient and reliable electric vehicle charging for the realisation of a true sustainable transportation landscape. Sci. Rep..

[38] Panda S., Mohanty S., Rout P.K., Sahu B.K., Parida S.M., Samanta I.S., Bajaj M., Piecha M., Blazek V., Prokop L. (2023). A comprehensive review on demand side management and market design for renewable energy support and integration. Energy Rep..

[39] Mohanty S., Panda S., Parida S.M., Rout P.K., Sahu B.K., Bajaj M., Zawbaa H.M., Kumar N.M., Kamel S. (2022). Demand side management of electric vehicles in smart grids: A survey on strategies, challenges, modeling, and optimization. Energy Rep..

[40] Panda S., Mohanty S., Rout P.K., Sahu B.K., Bajaj M., Zawbaa H.M., Kamel S. (2022). Residential Demand Side Management model, optimization and future perspective: A review. Energy Rep..

[41] Davoudkhani I.F., Zare P., Shenava S.J.S., Abdelaziz A.Y., Bajaj M., Tuka M.B. (2024). Maiden application of mountaineering team-based optimization algorithm optimized 1PD-PI controller for load frequency control in islanded microgrid with renewable energy sources. Sci. Rep..

[42] R Singh A., Kumar R.S., Bajaj M., Khadse C.B., Zaitsev I. (2024). Machine learning-based energy management and power forecasting in grid-connected microgrids with multiple distributed energy sources. Sci. Rep..

[43] Karthik N., Rajagopalan A., Bajaj M., Medhi P., Kanimozhi R., Blazek V., Prokop L. (2024). Chaotic self-adaptive sine cosine multi-objective optimization algorithm to solve microgrid optimal energy scheduling problems. Sci. Rep..

[44] Nadimuthu L.P.R., Victor K., Bajaj M., Tuka M.B. (2024). Feasibility of renewable energy microgrids with vehicle-to-grid technology for smart villages: A case study from India. Results Eng..

[45] Panda S., Samanta I.S., Rout P.K., Sahu B.K., Bajaj M., Blazek V., Prokop L., Misak S. (2024). Priority-based scheduling in residential energy management systems integrated with renewable sources using adaptive salp swarm algorithm. Results Eng..

[46] Nagarajan K., Rajagopalan A., Bajaj M., Raju V., Blazek V. (2025). Enhanced wombat optimization algorithm for multi-objective optimal power flow in renewable energy and electric vehicle integrated systems. Results Eng..

[47] Singh A.R., Dey B., Bajaj M., Kadiwala S., Kumar R.S., Dutta S., Zaitsev I. (2025). Advanced microgrid optimization using price-elastic demand response and greedy rat swarm optimization for economic and environmental efficiency. Sci. Rep..

[48] Ma K., Yang J., Liu P. (2020). Relaying-assisted communications for demand response in smart grid: Cost modeling, game strategies, and algorithms. IEEE J. Sel. Area. Commun..

[49] Yang J., Xu W., Ma K., Li C. (2023). A three-stage multi-energy trading strategy based on P2P trading mode. IEEE Trans. Sustain. Energy.

[50] Li L., Sun Y., Han Y., Chen W. (2024). Seasonal hydrogen energy storage sizing: Two-stage economic-safety optimization for integrated energy systems in northwest China. iScience.

[51] Zhang H., Yu C., Zeng M., Ye T., Yue D., Dou C., Xie X., Hancke G.P. (2024). Homomorphic Encryption Based Resilient Distributed Energy Management Under Cyber-Attack of Micro-Grid With Event-Triggered Mechanism. IEEE Trans. Smart Grid.

[52] Zhang H., Chen Z., Yu C., Yue D., Xie X., Hancke G.P. (2024). Event-Trigger-Based Resilient Distributed Energy Management Against FDI and DoS Attack of Cyber–Physical System of Smart Grid. IEEE Trans. Syst. Man Cybern. Syst..

[53] Zhang H., Yue D., Dou C., Hancke G.P. (2023). PBI based multi-objective optimization via deep reinforcement elite learning strategy for micro-grid dispatch with frequency dynamics. IEEE Trans. Power Syst..

[54] Chen H., Yang S., Wu H., Song J., Shui S. (2025). Advanced hierarchical energy optimization strategy for integrated electricity-heat-ammonia microgrid clusters in distribution network. Int. J. Hydrogen Energy.

[55] Deng X., Zhang Y., Jiang Y., Zhang Y., Qi H. (2024). A novel operation method for renewable building by combining distributed DC energy system and deep reinforcement learning. Appl. Energy.

[56] Zhang Y., Han X., Wei T., Zhao X., Zhang Y. (2023). Techno-environmental-economical performance of allocating multiple energy storage resources for multi-scale and multi-type urban forms towards low carbon district. Sustain. Cities Soc..

[57] Feng J., Yao Y., Liu Z. (2024). Developing an optimal building strategy for electric vehicle charging stations: automaker role. Environ. Dev. Sustain..

[58] Alhasnawi B.N., Zanker M., Bureš V. (2024). A smart electricity markets for a decarbonized microgrid system. Electr. Eng..

[59] Naji Alhasnawi B., Jasim B.H., Naji Alhasnawi A., Hussain F.F.K., Homod R.Z., Hasan H.A., Ibrahim Khalaf O., Abbassi R., Bazooyar B., Zanker M. (2024). A novel efficient energy optimization in smart urban buildings based on optimal demand side management. Energy Strategy Rev..

[60] Alhasnawi B.N., Jasim B.H., Sedhom B.E., Guerrero J.M. (2023). A new communication platform for smart EMS using a mixed-integer-linear-programming. Energy Syst..

[61] Alhasnawi B.N., Almutoki S.M.M., Hussain F.F.K., Harrison A., Bazooyar B., Zanker M., Bureš V. (2024). A new methodology for reducing carbon emissions using multi-renewable energy systems and artificial intelligence. Sustain. Cities Soc..

[62] Alhasnawi B.N., Mohammed N.A., Jasim B.H., Almutoki S.M.M., Hathal H.M., Mandeel T.H., Jasim A.M., Abbassi R., Bureš V., Sedhom B.E. (2024). Optimal loads scheduling using the intelligent optimization approach. AIP Conf. Proc..

[63] Bakhshaei P., Askarzadeh A., Arababadi R. (2021). Operation optimization of a grid-connected photovoltaic/pumped hydro storage considering demand response program by an improved crow search algorithm. J. Energy Storage.

[64] Alamir N., Kamel S., Megahed T.F., Hori M., Abdelkader S.M. (2023). Developing hybrid demand response technique for energy management in microgrid based on pelican optimization algorithm. Elec. Power Syst. Res..

[65] Datta J., Das D. (2023). Energy management study of interconnected microgrids considering pricing strategy under the stochastic impacts of correlated renewables. IEEE Syst. J..

[66] Datta J., Das D. (2023). Energy management of multi-microgrids with renewables and electric vehicles considering price-elasticity based demand response: A bi-level hybrid optimization approach. Sustain. Cities Soc..

[67] Kujur S., Dubey H.M., Salkuti S.R. (2023). Demand response management of a residential microgrid using chaotic aquila optimization. Sustainability.

[68] Chakraborty A., Ray S. (2024). Multi-objective operational cost management with minimum net emission of a smart microgrid. Elec. Power Compon. Syst..

[69] Chakraborty A., Ray S. (2024). Multi-objective energy management using a smart charging technique of a microgrid with the charging impact of plug-in hybrid electric vehicles. Sustain. Cities Soc..

[70] El-Bayeh C.Z., Alzaareer K., Aldaoudeyeh A.M.I., Brahmi B., Zellagui M. (2021). Charging and discharging strategies of electric vehicles: A survey. World Electr. Veh. J..

[71] Venkatesh B., Sankaramurthy P., Chokkalingam B., Mihet-Popa L. (2022). Managing the demand in a micro grid based on load shifting with controllable devices using hybrid WFS2ACSO technique. Energies.

[72] López K.L., Gagné C., Gardner M.A. (2018). Demand-side management using deep learning for smart charging of electric vehicles. IEEE Trans. Smart Grid.

[73] Sankaramurthy P., Chokkalingam B., Padmanaban S., Leonowicz Z., Adedayo Y. (2019). Rescheduling of generators with pumped hydro storage units to relieve congestion incorporating flower pollination optimization. Energies.

[74] Chakraborty A., Ray S. (2024). Microgrid operational energy management with plug-in hybrid electric vehicles charging demand. Electr. Eng..

[75] Ahmad F., Bilal M. (2024). Allocation of plug-in electric vehicle charging station with integrated solar powered distributed generation using an adaptive particle swarm optimization. Electr. Eng..

[76] Dey B., Dutta S., Garcia Marquez F.P. (2023). Intelligent demand side management for exhaustive techno-economic analysis of microgrid system. Sustainability.

[77] Chhual Singh T., Dey B., Rao K.S., Bhattacharyya B. (2023). Carbon constrained economic dispatch of a microgrid system based on different grid pricing strategies considering uncertainty. Environ. Prog. Sustain. Energy.

[78] Dey B., Misra S., Garcia Marquez F.P. (2023). Microgrid system energy management with demand response program for clean and economical operation. Appl. Energy.

[79] Dey B., Misra S., Chhualsingh T., Sahoo A.K., Singh A.R. (2024). A hybrid metaheuristic approach to solve grid centric cleaner economic energy management of microgrid systems. J. Clean. Prod..

[80] Misra S., Panigrahi P.K., Ghosh S., Dey B. (2024). A metaheuristic approach to compare different combined economic emission dispatch methods involving load shifting policy. Environ. Dev. Sustain..

